# Supplementary material for: Delphi studies in social and health sciences—Recommendations for an interdisciplinary standardized reporting (DELPHISTAR). Results of a Delphi study
Source: PLoS One. 2024 Aug 26;19(8):e0304651. doi: 10.1371/journal.pone.0304651 (PMC11346927; doi:10.1371/journal.pone.0304651)
Supplement: S2 File — (ZIP) [file pone.0304651.s002.zip › S2 File/DELPHISTAR_questionnaire_1.pdf]

Delphi studies in social and health sciences – recommendations for an interdisciplinary standardized reporting (DELPHISTAR)

Questionnaire for the first Delphi round

From: Niederberger, M.; Spranger, J. Deckert, S.; Hirt, J.; Homberg, A.; Köberich, S.; Kuhn, R.: Rommel, A.; Sonnberger, M. and the DEWISS network. Delphi studies in social and health sciences – recommendations for an interdisciplinary standardized reporting (DELPHISTAR). Results of a Delphi study.

More information at OSF (https://osf.io/gc4jk) and DEWISS (https://delphi.ph-gmuend.de/)

Note

We wish to point out that the questionnaire was sent out online using Unipark survey software. It is impossible to retain the exact formatting of the online version when converting it into a PDF file, which is why there may be differences in appearance between the two. The PDF version was created via Unipark.

Furthermore, the publication by Niederberger et al. titled "Delphi studies in social and health sciences – recommendations for an interdisciplinary standardized reporting (DELPHISTAR). Results of a Delphi study" focuses only on the questionnaire items connected with developing the reporting guideline. In addition to these, there were at the beginning of the survey two other topic blocks that were rated by the experts. This was not part of the paper. The questions and data regarding these two other topic blocks are still included in the questionnaire and dataset.

If there are any questions, please contact:

Prof. Dr. Marlen Niederberger
E-mail: marlen.niederberger(at)ph-gmuend.de
Department of Research Methods in Health Promotion and Prevention
Institute for Health Sciences, University of Education Schwäbisch Gmünd,
Oberbettringer Strasse 200, 73525 Schwäbisch Gmünd, Germany

DELPHISTAR
Delphi studies in health and social sciences – recommendations for a standardized reporting

Note

Dear experts, we have formulated the questionnaire in English. It is possible that the questionnaire is automatically translated into your national language due to your browser settings. Unfortunately, this may result in translation errors. To ensure the correctness of the content, we ask you to switch off automatic translation via the browser.

In Google Chrome you can change this under Settings, Advanced, Languages.

In Firefox, you will find the setting under the "Translate Web Pages" add-on (if installed).

In Microsoft Edge you will find the selection under Settings, Languages or you will be directly shown a menu for selection where you can decline the translation.

DELPHISTAR
Delphi studies in health and social sciences – recommendations for a standardized reporting

Block I: Characteristics of a Delphi Procedure

This chapter involves identifying the important characteristics of Delphi studies. The goal is to find a definition which applies to the different Delphi types, variations and modifications and is equally accepted and used in the health and social sciences. The reporting guideline will refer to this definition.

V1: In your opinion, how important or unimportant are the following characteristics for a Delphi Study?

Different characteristics that define a Delphi procedure are discussed in the methods literature (literature references are at the end of this page). Please evaluate the importance of each characteristic for a Delphi study. Please respond with a “1” if you consider the characteristic to be very unimportant or with a “7” if you consider it to be very important. You can use the numbers in between to graduate the scale. You may also indicate that you cannot or do not wish to evaluate a particular item.

	1 very unimportant	2	3	4	5	6	7 very important	cannot evaluate this item
1.1 Survey of several people with specialized knowledge (e.g., operational knowledge, experiential knowledge, functional knowledge, contextual knowledge)	○	○	○	○	○	○	○	○
1.2 Structured group communication process	○	○	○	○	○	○	○	○
1.3 Option to remain anonymous or to give individual responses in a safe space	○	○	○	○	○	○	○	○

1.4 Carrying out at least two survey rounds or the option to respond at least two times	◯	◯	◯	◯	◯	◯	◯	◯
1.5 Feedback, the (interim) results are presented to the respondents starting after the second round	◯	◯	◯	◯	◯	◯	◯	◯
1.6 Quantitative questionnaire as survey instrument, supplemented with qualitative items as needed	◯	◯	◯	◯	◯	◯	◯	◯
1.7 Analysis of the questionnaire (for feedback and for final results) is focused on statistical measures (e.g., mean values, variance)	◯	◯	◯	◯	◯	◯	◯	◯

S1: How certain are you in responding to this question?

Please respond with a “1” if you are extremely uncertain or with a “7” if you are absolutely certain. You can use the numbers in between to graduate the scale.

◯ 1 extremely uncertain
◯ 2
◯ 3
◯ 4
◯ 5
◯ 6
◯ 7 absolutely certain

O1: Here you have the option to give reasons for your responses and to provide additional information.

Please use the text box below.

-

Literature

- Linstone, H.A. M. Turoff, M (1975): The delphi method, Addison-Wesley, MA.
- Niederberger, M.; Spranger, J. (2020): Delphi technique in health sciences: A Map, Front. Public Health 8, 1–10.
- Rowe, G.; Wright, G.; Bolger, F. (1991): Delphi, A reevaluation of research and theory. Technological Forecasting and Social Change (39/3), S.235-251.
- Turoff, M.; Linstone, H.A. (2002 Eds.): The Delphi Method: Techniques and Applications, Addison-Wesley, Boston.
- Von der Gracht, H.A. (2012): Consensus measurement in Delphi studies. Review and implications for future quality assurance. In: Technological Forecasting and Social Change, 79, S. 1525-1536.

V2: Although reporting guidelines have proven effective in the health sciences for creating standards and quality control regarding academic publications, there are, as of yet, no recognized multidisciplinary and interdisciplinary reporting guideline for Delphi studies.

In your view, how probable is the development of a reporting guideline for Delphi studies that is agreed upon by experts, takes into account the different variations, types and modifications, and has validity in the health and social sciences?

Please respond with a “1” if you view the development of a reporting guideline to be very unlikely or with a “7” if you view this as being very likely. You can use the numbers in between to graduate the scale. You may also indicate that you cannot or do not wish to evaluate this item.

- ◯ 1 very unlikely
- ◯ 2
- ◯ 3
- ◯ 4
- ◯ 5
- ◯ 6
- ◯ 7 very likely
- ◯ cannot evaluate this item

S2: How certain are you in responding to this question?

Please respond with a “1” if you are extremely uncertain or with a “7” if you are absolutely certain. You can use the numbers in between to graduate the scale.

- ◯ 1 extremely uncertain
- ◯ 2
- ◯ 3
- ◯ 4
- ◯ 5
- ◯ 6
- ◯ 7 absolutely certain

O2: Here you have the option to give reasons for your response and to provide additional information.

Please use the text box below.

- More information on reporting guidelines can be found at https://www.equator-network.org/

Block II: Reporting Guideline

In this chapter we present different topics with concrete suggestions for the aspects to be reported under a reporting guideline for Delphi studies. These topics and aspects are the result of a systematic review and a method review of existing reporting guidelines for Delphi studies. We kindly ask you to rate the importance of these aspects as they pertain to a reporting guideline for Delphi studies.

To answer these questions, we recommend that you use the reporting guideline (PDF) sent to you by email. It will give you an overview of the topics and concrete aspects.

Title and Abstract

V3: If Delphi studies and their results are reported, how important do you consider the following aspects of the "Title and Abstract"?

Please respond with a “1” if you view an aspect as very unimportant or with a “7” if you view it as being very important. You can use the numbers in between to graduate the scale. You may also indicate that you cannot or do not wish to evaluate a particular item.

	1 very unimportant	2	3	4	5	6	7 very important	cannot evaluate this item
3.a Identification as a Delphi procedure in the title	○	○	○	○	○	○	○	○
3.b Identification as a Delphi procedure in the abstract	○	○	○	○	○	○	○	○
3.c Structured abstract (e.g., background, method, results and discussion)	○	○	○	○	○	○	○	○

S3: How certain are you in responding to the topic "Title and Abstract" of the Reporting Guideline?

Please respond with a “1” if you are extremely uncertain or with a “7” if you are absolutely certain. You can use the numbers in between to graduate the scale.

- ◯ 1 extremely uncertain
- ◯ 2
- ◯ 3
- ◯ 4
- ◯ 5
- ◯ 6
- ◯ 7 absolutely certain

O3: In the following you have the option to give reasons for your responses and to provide additional information.

Please use the text box below.

-

Literature

- Niederberger, M.; Köberich, S. & DeWiss (2021). The Delphi Technique. EJCN. https://doi:10.1093/eurjcn/zvab05
- Niederberger, M.; Spranger, J. (2020): Delphi technique in health sciences: A Map, Front. Public Health 8, 1–10. https://doi.org/10.3389/fpubh.2020.00457.

Context

V4: If Delphi studies and their results are reported, how important do you consider the following aspects of the "Context - Section: Formal"?

Please respond with a “1” if you view an aspect as very unimportant or with a “7” if you view it as being very important. You can use the numbers in between to graduate the scale. You may also indicate that you cannot or do not wish to evaluate a particular item.

	1 very unimportant	2	3	4	5	6	7 very important	cannot evaluate this item
4.a Information about the sources of funding	◯	◯	◯	◯	◯	◯	◯	◯
4.b Information about the team of authors and/or researchers (e.g., discipline, institution)	◯	◯	◯	◯	◯	◯	◯	◯
4.c Information about the methods consulting	◯	◯	◯	◯	◯	◯	◯	◯
4.d Information about the project’s background	◯	◯	◯	◯	◯	◯	◯	◯
4.e Time of the Delphi study	◯	◯	◯	◯	◯	◯	◯	◯
4.f Information about the study protocol	◯	◯	◯	◯	◯	◯	◯	◯
4.g Information about the ethics vote	◯	◯	◯	◯	◯	◯	◯	◯
4.h Reference to additional information or materials about the project or Delphi study (e.g., online materials, Internet site)	◯	◯	◯	◯	◯	◯	◯	◯

Context

V5: If Delphi studies and their results are reported, how important do you consider the following aspects of the "Context - Section: Theory"?

Please respond with a “1” if you view an aspect as very unimportant or with a “7” if you view it as being very important. You can use the numbers in between to graduate the scale. You may also indicate that you cannot or do not wish to evaluate a particular item.

	1 very unimportant	2	3	4	5	6	7 very important	cannot evaluate this item
5.a Positioning within the philosophy of science (e.g., realistic, positivist, constructivist)	◯	◯	◯	◯	◯	◯	◯	◯
5.b Identification of the research paradigm (in particular, qualitative or quantitative)	◯	◯	◯	◯	◯	◯	◯	◯
5.c Statement of presuppositions (e.g., regarding potentially contradictory topics)	◯	◯	◯	◯	◯	◯	◯	◯

Context

V6: If Delphi studies and their results are reported, how important do you consider the following aspects of the topic "Context - Sektion: Content"?

Please respond with a “1” if you view an aspect as very unimportant or with a “7” if you view it as being very important. You can use the numbers in between to graduate the scale. You may also indicate that you cannot or do not wish to evaluate a particular item.

	1 very unimportant	2	3	4	5	6	7 very important	cannot evaluate this item
6.a Reflection on the relevance of the Delphi procedure as a topic, taking current research and the existing evidence base into account	◯	◯	◯	◯	◯	◯	◯	◯
6.b Reflection on the relevance of the Delphi procedure as a topic, taking social developments and innovations into account (e.g., the Covid-19 pandemic)	◯	◯	◯	◯	◯	◯	◯	◯
6.c Justification of the chosen method (Delphi procedure) to answer the research question	◯	◯	◯	◯	◯	◯	◯	◯
6.d Aim of the Delphi procedure (e.g., consensus, forecasting)	◯	◯	◯	◯	◯	◯	◯	◯

6.e Information about the combination of the Delphi procedure with other studies (e.g., mixed-methods design, triangulation)	◯	◯	◯	◯	◯	◯	◯	◯

S4-6: How certain are you in responding to the topic "Context" of the Reporting Guideline?

Please respond with a “1” if you are extremely uncertain or with a “7” if you are absolutely certain. You can use the numbers in between to graduate the scale.

- ◯ 1 extremely uncertain
- ◯ 2
- ◯ 3
- ◯ 4
- ◯ 5
- ◯ 6
- ◯ 7 absolutely certain

O4-6: In the following you have the option to give reasons for your responses and to provide additional information.

Please use the text box below.

-

Method

V7: If Delphi studies and their results are reported, how important do you consider the following aspects of the "Method - Section: Body of knowledge & Integration of knowledge"?

Please respond with a "1" if you view an aspect as very unimportant or with a "7" if you view it as being very important. You can use the numbers in between to graduate the scale. You may also indicate that you cannot or do not wish to evaluate a particular item.

	1 very unimportant	2	3	4	5	6	7 very important	cannot evaluate this item
7.a Identification and elucidation of relevant expertise, spheres of experience, and perspectives (e.g., theory, practice, affected groups, disciplines)	◯	◯	◯	◯	◯	◯	◯	◯
7.b Handling of knowledge, expertise and perspectives which are missing or have been deliberately not integrated	◯	◯	◯	◯	◯	◯	◯	◯
7.c Basic definition of expert (1)	◯	◯	◯	◯	◯	◯	◯	◯

(1) Previous reference in questionnaire: For us, "experts" are the participants; this can be people from academia, practice, or representatives of lived experience (e.g., patients, family members).

Method

V8: If Delphi studies and their results are reported, how important do you consider the following aspects of the "Method - Section: Delphi variations"?

Please respond with a “1” if you view an aspect as very unimportant or with a “7” if you view it as being very important. You can use the numbers in between to graduate the scale. You may also indicate that you cannot or do not wish to evaluate a particular item.

	1 very unimportant	2	3	4	5	6	7 very important	cannot evaluate this item
8.a Identification of potential preliminary studies for the Delphi procedure (e.g., qualitative expert interviews, literature reviews)	○	○	○	○	○	○	○	○
8.b Identification of the type of Delphi procedure and potential modifications (e.g., classic Delphi, real-time Delphi, group Delphi)	○	○	○	○	○	○	○	○
8.c Justification of the Delphi variation and modifications, including during the Delphi process, if applicable	○	○	○	○	○	○	○	○

Method

V9: If Delphi studies and their results are reported, how important do you consider the following aspects of the "Method - Section: Sample of experts"?

Please respond with a “1” if you view an aspect as very unimportant or with a “7” if you view it as being very important. You can use the numbers in between to graduate the scale. You may also indicate that you cannot or do not wish to evaluate a particular item.

	1 very unimportant	2	3	4	5	6	7 very important	cannot evaluate this item
9.a Selection criteria for the experts (per round if there are different expert groups)	◯	◯	◯	◯	◯	◯	◯	◯
9.b Identification of the experts	◯	◯	◯	◯	◯	◯	◯	◯
9.c Information about recruiting and any subsequent recruiting of experts	◯	◯	◯	◯	◯	◯	◯	◯
9.d Number of experts per round (both invited and participating)	◯	◯	◯	◯	◯	◯	◯	◯
9.e Information about how refusals and dropouts are handled	◯	◯	◯	◯	◯	◯	◯	◯
9.f Information about data protection regarding the experts	◯	◯	◯	◯	◯	◯	◯	◯
9.g Anonymity of the experts	◯	◯	◯	◯	◯	◯	◯	◯
9.h Information about the experts' sociodemographics per round	◯	◯	◯	◯	◯	◯	◯	◯

9.i Information about expert competency per round	◯	◯	◯	◯	◯	◯	◯	◯

Method

V10: If Delphi studies and their results are reported, how important do you consider the following aspects of the "Method - Section: Survey"?

Please respond with a “1” if you view an aspect as very unimportant or with a “7” if you view it as being very important. You can use the numbers in between to graduate the scale. You may also indicate that you cannot or do not wish to evaluate a particular item.

	1 very unimportant	2	3	4	5	6	7 very important	cannot evaluate this item
10.a Elucidation of the content development for the questionnaire (2)	◯	◯	◯	◯	◯	◯	◯	◯
10.b Structure of the questionnaire (e.g., sections, organization)	◯	◯	◯	◯	◯	◯	◯	◯
10.c Number of items (open, closed, hybrid)	◯	◯	◯	◯	◯	◯	◯	◯
10.d Reference to additional integrated materials or information (e.g., info boxes)	◯	◯	◯	◯	◯	◯	◯	◯
10.e Information about and justification of the types of scales used	◯	◯	◯	◯	◯	◯	◯	◯
10.f Information about the graphic design of the questionnaire (e.g., use of figures)	◯	◯	◯	◯	◯	◯	◯	◯
10.g Information about the validity of the items/scales	◯	◯	◯	◯	◯	◯	◯	◯

10.h Information about the query regarding the experts' degree of certainty or competency	◯	◯	◯	◯	◯	◯	◯	◯
10.i Information about the pretest for the questionnaire	◯	◯	◯	◯	◯	◯	◯	◯
10.j Length of time to fill out the questionnaire per round	◯	◯	◯	◯	◯	◯	◯	◯
10.k Information about the software used for the survey (e.g., soscisurvey, e-delphi)	◯	◯	◯	◯	◯	◯	◯	◯

(2) Note: We use the term “questionnaire” for the survey instrument regardless of whether quantitative or qualitative items are integrated or weighted.

Method

V11: If Delphi studies and their results are reported, how important do you consider the following aspects of the "Method - Section: Delphi rounds"?

Please respond with a "1" if you view an aspect as very unimportant or with a "7" if you view it as being very important. You can use the numbers in between to graduate the scale. You may also indicate that you cannot or do not wish to evaluate a particular item.

	1 very unimportant	2	3	4	5	6	7 very important	cannot evaluate this item
11.a Number of Delphi rounds	○	○	○	○	○	○	○	○
11.b Information about the aims of the individual Delphi rounds	○	○	○	○	○	○	○	○
11.c Disclosure and justification of the criterion for discontinuation	○	○	○	○	○	○	○	○

DELPHISTAR
Delphi studies in health and social sciences – recommendations for a standardized reporting

Method

V12: If Delphi studies and their results are reported, how important do you consider the following aspects of the "Method - Section: Feedback"?

Please respond with a “1” if you view an aspect as very unimportant or with a “7” if you view it as being very important. You can use the numbers in between to graduate the scale. You may also indicate that you cannot or do not wish to evaluate a particular item.

	1 very unimportant	2	3	4	5	6	7 very important	cannot evaluate this item
12.a Information about what data was reported back per round	○	○	○	○	○	○	○	○
12.b Information about the form of the feedback (e.g., statistical, graphical)	○	○	○	○	○	○	○	○
12.c Information about any differentiated feedback (e.g., according to expert group)	○	○	○	○	○	○	○	○

S7-12: How certain are you in responding to the topic "Method" of the Reporting Guideline?

Please respond with a “1” if you are extremely uncertain or with a “7” if you are absolutely certain. You can use the numbers in between to graduate the scale.

- ◯ 1 extremely uncertain
- ◯ 2
- ◯ 3
- ◯ 4
- ◯ 5
- ◯ 6
- ◯ 7 absolutely certain

O7-12: In the following you have the option to give reasons for your responses and to provide additional information.

Please use the text box below.

-

Data analysis and results

V13: If Delphi studies and their results are reported, how important do you consider the following aspects of the "Data analysis and results - Section: Data analysis"?

Please respond with a “1” if you view an aspect as very unimportant or with a “7” if you view it as being very important. You can use the numbers in between to graduate the scale. You may also indicate that you cannot or do not wish to evaluate a particular item.

	1 very unimportant	2	3	4	5	6	7 very important	cannot evaluate this item
13.a. Disclosure of the quantitative and qualitative analytical strategy	◯	◯	◯	◯	◯	◯	◯	◯
13.b. Information about the software used for analysis (e.g., SPSS, R, MAXQDA)	◯	◯	◯	◯	◯	◯	◯	◯
13.c. Definition and measurement of consensus	◯	◯	◯	◯	◯	◯	◯	◯
13.d. Information on group-specific analysis or weighting of experts (e.g., theory vs. practice, discipline-specific analysis)	◯	◯	◯	◯	◯	◯	◯	◯

Data analysis and results

V14: If Delphi studies and their results are reported, how important do you consider the following aspects of the "Data analysis and results - Section: Delphi process"?

Please respond with a “1” if you view an aspect as very unimportant or with a “7” if you view it as being very important. You can use the numbers in between to graduate the scale. You may also indicate that you cannot or do not wish to evaluate a particular item.

	1 very unimportant	2	3	4	5	6	7 very important	cannot evaluate this item
14.a Illustration of the Delphi process (e.g., in a flow chart)	○	○	○	○	○	○	○	○
14.b Information about special aspects during the Delphi process (e.g., deviations from the intended approach with justification)	○	○	○	○	○	○	○	○

Data analysis and results

V15: If Delphi studies and their results are reported, how important do you consider the following aspects of the "Data analysis and results - Section: Results"?

Please respond with a “1” if you view an aspect as very unimportant or with a “7” if you view it as being very important. You can use the numbers in between to graduate the scale. You may also indicate that you cannot or do not wish to evaluate a particular item.

	1 very unimportant	2	3	4	5	6	7 very important	cannot evaluate this item
15.a Presentation of the results for each Delphi round and the final results	◯	◯	◯	◯	◯	◯	◯	◯
15.b Information about how dissent and unclear results were handled	◯	◯	◯	◯	◯	◯	◯	◯

S13-15: How certain are you in responding to the topic "Data analysis and results" of the Reporting Guideline?

Please respond with a “1” if you are extremely uncertain or with a “7” if you are absolutely certain. You can use the numbers in between to graduate the scale.

- ◯ 1 extremely uncertain
- ◯ 2
- ◯ 3
- ◯ 4
- ◯ 5
- ◯ 6
- ◯ 7 absolutely certain

O13-15: In the following you have the option to give reasons for your responses and to provide additional information.

Please use the text box below.

-

Discussion and Dissemination

V16: If Delphi studies and their results are reported, how important do you consider the following aspects of the "Discussion and Dissemination - Section: Quality of findings"?

Please respond with a “1” if you view an aspect as very unimportant or with a “7” if you view it as being very important. You can use the numbers in between to graduate the scale. You may also indicate that you cannot or do not wish to evaluate a particular item.

	1 very unimportant	2	3	4	5	6	7 very important	cannot evaluate this item
16.a Validity of the results (e.g., transferability of the findings)	○	○	○	○	○	○	○	○
16.b Reliability of the results (e.g., when analyzing open-ended responses, intersubjectivity)	○	○	○	○	○	○	○	○
16.c Validation of the findings (e.g., communicative feedback for the respondents)	○	○	○	○	○	○	○	○
16.d Reflection on potential limitations (e.g., distortion, skewing, bias)	○	○	○	○	○	○	○	○

Discussion and Dissemination

V17: If Delphi studies and their results are reported, how important do you consider the following aspects of the "Discussion and Dissemination - Section: Dissemination"?

Please respond with a “1” if you view an aspect as very unimportant or with a “7” if you view it as being very important. You can use the numbers in between to graduate the scale. You may also indicate that you cannot or do not wish to evaluate a particular item.

	1 very unimportant	2	3	4	5	6	7 very important	cannot evaluate this item
17.a Availability of the dataset	○	○	○	○	○	○	○	○
17.b Information about whether the results were made available to the experts	○	○	○	○	○	○	○	○
17.c Accessibility of the results for interested members of the public	○	○	○	○	○	○	○	○
17.d Information about further use of the results	○	○	○	○	○	○	○	○

S16-17: How certain are you in responding to the topic "Discussion and Dissemination" of the Reporting Guideline?

Please respond with a “1” if you are extremely uncertain or with a “7” if you are absolutely certain. You can use the numbers in between to graduate the scale.

- ◯ 1 extremely uncertain
- ◯ 2
- ◯ 3
- ◯ 4
- ◯ 5
- ◯ 6
- ◯ 7 absolutely certain

O16-17: In the following you have the option to give reasons for your responses and to provide additional information.

Please use the text box below.

-

Block III: Personal Questions

P1: Which discipline do you feel you belong to the most?

Please mark the one that matches most closely.

- ◯ Humanities
- ◯ Health science
- ◯ Natural science
- ◯ Engineering science
- ◯ Other, specifically:

____

P2: In which country are you regularly employed at present?

Please mark the country that matches most closely.

- ◯ Argentina
- ◯ Australia
- ◯ Belgium
- ◯ Brazil
- ◯ Chile
- ◯ China
- ◯ Denmark
- ◯ Germany
- ◯ England/UK
- ◯ Finland
- ◯ France
- ◯ Greece
- ◯ India
- ◯ Iran

- ◯ Ireland
- ◯ Israel
- ◯ Italy
- ◯ Japan
- ◯ Canada
- ◯ Colombia
- ◯ Korea
- ◯ Lebanon
- ◯ Malaysia
- ◯ Mexico
- ◯ New Zealand
- ◯ Netherlands
- ◯ Nigeria
- ◯ Norway
- ◯ Austria
- ◯ Palestine
- ◯ Poland
- ◯ Portugal
- ◯ Russia
- ◯ Sweden
- ◯ Switzerland
- ◯ Serbia
- ◯ Singapore
- ◯ Slovenia
- ◯ Spain
- ◯ South Africa
- ◯ South Korea
- ◯ Taiwan
- ◯ Thailand
- ◯ Tunisia

◯ Turkey

◯ Hungary

◯ USA

◯ Other, specifically:

____

P3: How many Delphi studies have you already participated in (as a leader, a consultant or person carrying out the study, not as a respondent)?

Please state the number. If you do not know the exact number, please estimate how many.

____

P4: How many <u>Delphi publications</u> (with or without peer review process) have you already participated in?

Please state the number. If you do not know the exact number, please estimate how many.

____

P5: In which year did you first encounter a Delphi procedure?

If you do not know the exact year, please estimate when.

____

P6: How well do you assess your ability to apply the different Delphi variations?

Please mark the one that matches most closely.

	1 absolutely no ability	2	3	4	5	6	7 excellent ability	I don't know
Classic Delphi	◯	◯	◯	◯	◯	◯	◯	◯
Real-time Delphi	◯	◯	◯	◯	◯	◯	◯	◯
Group Delphi	◯	◯	◯	◯	◯	◯	◯	◯
Policy Delphi	◯	◯	◯	◯	◯	◯	◯	◯
Argumentative Delphi	◯	◯	◯	◯	◯	◯	◯	◯
Deliberative Delphi	◯	◯	◯	◯	◯	◯	◯	◯
Fuzzy Delphi	◯	◯	◯	◯	◯	◯	◯	◯
Other Delphi variation, specifically: ______________________________ -	◯	◯	◯	◯	◯	◯	◯	◯

P7: Which of the following profiles best describes your expertise on Delphi studies?

Please mark the one that matches most closely.

- ◯ Delphi beginner
- ◯ Delphi user
- ◯ Delphi expert

P8: How would you best describe your response behavior as you filled out the questionnaire?

Please mark the best description.

- ◯ Considered
- ◯ Intuitive
- ◯ Sometimes considered/sometimes intuitive
- ◯ I can't say

In which language did you answer the questionnaire?

Please select.

- ◯ English
- ◯ In another language.
