# Supplementary material for: Delphi studies in social and health sciences—Recommendations for an interdisciplinary standardized reporting (DELPHISTAR). Results of a Delphi study
Source: PLoS One. 2024 Aug 26;19(8):e0304651. doi: 10.1371/journal.pone.0304651 (PMC11346927; doi:10.1371/journal.pone.0304651)
Supplement: S2 File — (ZIP) [file pone.0304651.s002.zip › S2 File/DELPHISTAR_questionnaire_2.pdf]

Delphi studies in social and health sciences – recommendations for an interdisciplinary standardized reporting (DELPHISTAR)

Questionnaire for the second Delphi round

From: Niederberger, M.; Spranger, J. Deckert, S.; Hirt, J.; Homberg, A.; Köberich, S.; Kuhn, R.: Rommel, A.; Sonnberger, M. and the DEWISS network. Delphi studies in social and health sciences – recommendations for an interdisciplinary standardized reporting (DELPHISTAR). Results of a Delphi study.

More information at OSF (https://osf.io/gc4jk) and DEWISS (https://delphi.ph-gmuend.de/)

Note

We wish to point out that the questionnaire was sent out online using Unipark survey software. It is impossible to retain the exact formatting of the online version when converting it into a PDF file, which is why there may be differences in appearance between the two. The PDF version was created via Unipark.

Furthermore, the publication by Niederberger et al. titled "Delphi studies in social and health sciences – recommendations for an interdisciplinary standardized reporting (DELPHISTAR). Results of a Delphi study" focuses only on the questionnaire items connected with developing the reporting guideline. In addition to these, there were at the beginning of the survey two other topic blocks that were rated by the experts. This was not part of the paper. The questions and data regarding these two other topic blocks are still included in the questionnaire and dataset.

If there are any questions, please contact:

Prof. Dr. Marlen Niederberger
E-mail: marlen.niederberger(at)ph-gmuend.de
Department of Research Methods in Health Promotion and Prevention
Institute for Health Sciences, University of Education Schwäbisch Gmünd,
Oberbettringer Strasse 200, 73525 Schwäbisch Gmünd, Germany

DELPHISTAR
Delphi studies in health and social sciences – recommendations for a standardized reporting

Note

Dear experts, we have formulated the questionnaire in English. It is possible that the questionnaire is automatically translated into your national language due to your browser settings. Unfortunately, this may result in translation errors. To ensure the correctness of the content, we ask you to switch off automatic translation via the browser.
In Google Chrome you can change this under Settings, Advanced, Languages.
In Firefox, you will find the setting under the "Translate Web Pages" add-on (if installed).
In Microsoft Edge you will find the selection under Settings, Languages or you will be directly shown a menu for selection where you can decline the translation.

Remarks on the evaluation of the first Delphi round

Questions that are in consensus according to our definition are no longer shown in this Delphi round. We used the following consensus definition:

- For seven-point rating scales: at least 75% of the answers are on scale values 6 and 7 (agreement) and the standard deviation is at most 1.0, i.e., there is a relatively low dispersion of the answers.

The **open-ended responses** were qualitatively evaluated using content analysis. The focus was on the range of arguments rather than quantification. The arguments are listed in this round. They do not reflect the opinion of the research team.

Items that we define as consensus are no longer evaluated in this Delphi round, but are still listed if necessary to understand the questions that follow. You will also **see your individual response** from the first Delphi round for each item.

Statistical terms and abbreviations in the questionnaire

- **n**: number of cases
- **Mean**: „average“
- **Standard deviation (S)**: average deviation of the observed values from the mean value

DELPHISTAR
Delphi studies in health and social sciences – recommendations for a standardized reporting

Block I: Characteristics of a Delphi Procedure

This section involves identifying the important characteristics of Delphi studies. The goal is to find a definition which applies to the different Delphi types, variations and modifications and is equally accepted and used in the health and social sciences. The reporting guideline will refer to this definition.

V1: In your opinion, how important or unimportant are the following characteristics for a Delphi Study?

Different characteristics that define a Delphi procedure are discussed in the methods literature (literature references are at the end of this page). Please evaluate the importance of each characteristic for a Delphi study.

Please respond with a “1” if you consider the characteristic to be very unimportant or with a “7” if you consider it to be very important. You can use the numbers in between to graduate the scale. You may also indicate that you cannot or do not wish to evaluate a particular item. In the brackets you will find the results from the first Delphi round.

These items were in consensus in the first Delphi-Round.

V1.1 Survey of several people with specialized knowledge (e.g., operational knowledge, experiential knowledge, functional knowledge, contextual knowledge)

V1.5 Feedback, the (interim) results are presented to the respondents starting the second round

	1 very unimportant	2	3	4	5	6	7 very important	cannot evaluate this item
1.2 Structured group communication process (62% agreement on importance (6 and 7); Mean=5.6; S=1.6; n=90)	○	○	○	○	○	○	○	○

1.3 Option to remain anonymous or to give individual responses in a safe space (59% agreement on importance (6 and 7); Mean=5.4; S=1.9; n=91)	○	○	○	○	○	○	○	○
1.4 Carrying out at least two survey rounds or the option to respond at least two times (71% agreement on importance (6 and 7); Mean=6.1; S=1.3; n=91)	○	○	○	○	○	○	○	○
1.6 Quantitative questionnaire as survey instrument, supplemented with qualitative items as needed (67% agreement on importance (6 and 7); Mean=5.7; S=1.3; n=90)	○	○	○	○	○	○	○	○
1.7 Analysis of the questionnaire (for feedback and for final results) is focused on statistical measures (e.g., mean values, variance) (53% agreement on importance (6 and 7); Mean=5.3; S=1.7; n=90)	○	○	○	○	○	○	○	○

S1: We would now be interested in your personal assessment of your certainty of judgement. How certain are you in responding to this question?

Please respond with a “1” if you are extremely uncertain or with a “7” if you are absolutely certain. You can use the numbers in between to graduate the scale.

- ◯ 1 extremely uncertain
- ◯ 2
- ◯ 3
- ◯ 4
- ◯ 5
- ◯ 6
- ◯ 7 absolutely certain

O1: In the following you have the possibility to justify your answer and thus extend the list of arguments from the first Delphi round. If you do not have a new argument, you can skip the text field.

Please use the text box below. Please note: The text box is limited to 300 characters!

Open comments of the first Delphi round, sorted by topics

Definition criteria

- Variance should be pointed out.
- Definition criteria omit some of the newer developments in Delphi such as the potential for real-time Delphi surveys

Communication process

- Process is needed to obtain independent & deliberative input.
- Group communication is not absolutely necessary. Communication is often done individually by individuals.

Anonymity

- Complete anonymity is concerning.
- … is not necessary.
- … is particularly important for controversial topics.

Number of rounds

- Sometimes three to four rounds are needed. These processes should not be terminated because of the fulfillment of two rounds.
- … depends on the pre-work completed (e.g. literature search) and the levels of consensus.
- … depends strongly on Delphi setting and the quality of questionnaire developed during the process. A strict indication of the number of rounds should be avoided.

Qualitative versus quantitative Paradigm

- A qualitative questionnaire is qualitative research, not Delphi.
- Quantitative components and statistical analysis should predominate so that conclusions are more universal and evidence-based.
- An initial round may be all qualitative, but this should not be overused further on.
- Some topics do not lend themselves to quantitative analysis.
- Qualitative items can help to understand unclear definitions.
- Arguments and qualitative responses are key to the structured discussion sought in a Delphi. Ratings are only a tool to see how far you are.
- Several types of Delphi do not require numerical analysis, such as scenario generation Delphi or certain focus groups.
- All issues are priority and equally important to ensure the robustness of Delphi, as qualitative-quantitative model.

-

Literature

- Linstone, H.A. M. Turoff, M (1975): The delphi method, Addison-Wesley, MA.
- Niederberger, M.; Spranger, J. (2020): Delphi technique in health sciences: A Map, Front. Public Health 8, 1–10.
- Rowe, G.; Wright, G.; Bolger, F. (1991): Delphi, A reevaluation of research and theory. Technological Forecasting and Social Change (39/3), S.235-251.
- Turoff, M.; Linstone, H.A. (2002 Eds.): The Delphi Method: Techniques and Applications, Addison-Wesley, Boston.
- Von der Gracht, H.A. (2012): Consensus measurement in Delphi studies. Review and implications for future quality assurance. In: Technological Forecasting and Social Change, 79, S. 1525-1536.

V2: Although reporting guidelines have proven effective in the health sciences for establishing standards and quality control for academic publications, there are no reporting guidelines for Delphi studies yet that are recognized by different disciplines and that consider the different distinctive ways in which they may be conducted.

In your opinion, how likely is the development of an expert-agreed reporting guideline for Delphi studies valid for the social and health sciences in which the different variants, types and modifications are considered?

Please respond with a “1” if you view the development of a reporting guideline to be very unlikely or with a “7” if you view this as being very likely. You can use the numbers in between to graduate the scale. You may also indicate that you cannot or do not wish to evaluate this item.

Results of the first Delphi round: 59% agreement on 6 and 7; Mean=5.6; S=1.1; n=85

- ◯ 1 very unlikely
- ◯ 2
- ◯ 3
- ◯ 4
- ◯ 5
- ◯ 6
- ◯ 7 very likely
- ◯ cannot evaluate this item

S2: How certain are you in responding to this question?

Please respond with a “1” if you are extremely uncertain or with a “7” if you are absolutely certain. You can use the numbers in between to graduate the scale.

- ◯ 1 extremely uncertain
- ◯ 2
- ◯ 3
- ◯ 4
- ◯ 5
- ◯ 6
- ◯ 7 absolutely certain

O2: In the following you have the possibility to justify your answer and thus extend the list of arguments from the first Delphi round. If you do not have a new argument, you can skip the text field.

Please use the text box below. Please note: The text box is limited to 300 characters!

Open comments of the first Delphi round, sorted by topics

Concerns about consensus building

- Delphi studies have to be adapted for particular situations. There is not enough comparative research to prove that one method is better than another. The consensus view identified in these reporting guidelines will have extremely weak evidence and may be wrong.
- Validity in the health and social sciences is questionable as Delphi often generate consensus when there is less evidence. Consensus cannot always be reached by qualitative data; it is important to ensure that all arguments have been considered.

Transparency and regulation

- Reporting guidelines should address transparency in analysis and presentation of responses, and e.g., the selection of experts. People should stop calling iterative surveys seeking consensus Delphi studies.
- If the reporting is standardized, the Delphi protocols will also be regulated.
- It is important to define the criteria to support future studies and to ensure the credibility of these types of studies, even if it is difficult.
- As a scientific method, it should have a specific definition and boundary. With that principle, a guideline on application of any scientific method could be developed no matter how variable it is.
- Existing frameworks are well used indicating there is a need. (e.g., doi: 10.1177/0269216317690685)

No need for a reporting guideline

- Reporting guidelines already exist.
- Having gone a long time without a guideline, guideline development may not be a burning issue.
- Unclear why developing guidelines would improve the accuracy of Delphi results more than, say, conducting a meta-analysis of existing Delphi studies on the same or similar topics and looking for consistencies in the prediction.

Different Delphi applications

- There are applications in new and unexplored sectors that may reveal unique features.
- Flexibility to adapt to unique applications/situations is important
- There is no one size fits all format. Policy Delphi and regular Delphi absolutely need to be distinguished. The difference is extremely important.
- It is important to better understand the variety of types of Delphi, and reach consensus on appropriate methods.
- There are so many forms and interpretations of Delphi as used by different disciplines

Feasibility

- Developing a standardized procedure will be challenging due to many different opinions, but not impossible.
- Developing guidelines is certainly feasible.
- Developing possible models for the respective field of study, as is being done in built environment research, would be best and also feasible.

_ More information on reporting guidelines can be found at https://www.equator-network.org/

Block II: Reporting Guideline

In this chapter we present different topics with concrete suggestions for the aspects to be reported under a reporting guideline for Delphi studies. These topics and aspects are the result of a systematic review and a method review of existing reporting guidelines for Delphi studies. We kindly ask you to rate the importance of these aspects as they pertain to a reporting guideline for Delphi studies.

To answer these questions, we recommend that you use the reporting guideline (PDF) sent to you by email. It will give you an overview of the topics and concrete aspects. Only the items that were not in consensus in the first Delphi round are queried.

Title and Abstract

V3: If Delphi studies and their results are reported, how important do you consider the following aspects of the "Title and Abstract"?

Please respond with a “1” if you view an aspect as very unimportant or with a “7” if you view it as being very important. You can use the numbers in between to graduate the scale. You may also indicate that you cannot or do not wish to evaluate a particular item. In the brackets you will find the results from the first Delphi round.

	1 very unimportant	2	3	4	5	6	7 very important	cannot evaluate this item
3.a Identification as a Delphi procedure in the title (73% agreement on 6 and 7; Mean=5.9; S=1.6; n=91)	○	○	○	○	○	○	○	○
3.c Structured abstract (e.g., background, method, results and discussion) (73% agreement on 6 and 7; Mean=6; S=1.5; n=91)	○	○	○	○	○	○	○	○

S3: How certain are you in responding to the topic "Title and Abstract" of the Reporting Guideline?

Please respond with a “1” if you are extremely uncertain or with a “7” if you are absolutely certain. You can use the numbers in between to graduate the scale.

- ◯ 1 extremely uncertain
- ◯ 2
- ◯ 3
- ◯ 4
- ◯ 5
- ◯ 6
- ◯ 7 absolutely certain

O3: In the following you have the possibility to justify your answer and thus extend the list of arguments from the first Delphi round. If you do not have a new argument, you can skip the text field.

Please use the text box below. Please note: The text box is limited to 300 characters!

Open comments of the first Delphi round, sorted by topics

Dependence on journal guidelines

- Abstract structure is chosen by the journal.
- Structured abstracts are not supported by all journals, and are not appropriate for some qualitative studies.
- Sometimes the method cannot be stated in the title due to length restrictions. Then the specification in the abstract is absolutely necessary.

Labeling as a Delphi in the title and/or abstract

- … is essential.
- … is important for the reader to understand the subject.

- … is also standard items for major reporting guidelines (e.g., PRISMA, CONSORT, STROBE)
- … is entirely appropriate/essential for transparent reporting.
- … is important for the reader or researcher to know that this is a Delphi study just like a RCT or meta analysis.
- The title should give sufficient information to identify the Delphi. The abstract should clearly show the Delphi variant used.

Labeling as a Delphi in the title and/or abstract is not essential

- Details of methodology do not need to be in the title. The title could be just about the content of the paper. Especially if there are many methodological elements that cannot all be stated in the title.
- For example, if Delphi is only part of the method "mixed methods" can be used in the title.
- Having the term in the title and/or abstract is helpful for findability but otherwise not essential. This is essential for the methods section.
- Delphi means different things to different people. It is less important to use the word “Delphi” and more important to define exactly what was done so that others can replicate, or at least corroborate your findings.
- Title and abstracts may not always provide an excellent methodology

---

-

Literature

- Niederberger, M.; Spranger, J. (2020). Delphi technique in health sciences: A Map, Front. Public Health 8, 1–10. https://doi.org/10.3389/fpubh.2020.00457.
- Spranger, J.; Homberg, A.; Sonnberger, M.; & Niederberger, M. (2022). Reporting guidelines for Delphi techniques in health sciences: A methodological review. Z Evid Fortbild Qual Gesundhwes. *172*, 1–11. https://doi.org/10.1016/j.zefq.2022.04.025

Context

V4: If Delphi studies and their results are reported, how important do you consider the following aspects of the "Context - Section: Formal"?

Please respond with a “1” if you view an aspect as very unimportant or with a “7” if you view it as being very important. You can use the numbers in between to graduate the scale. You may also indicate that you cannot or do not wish to evaluate a particular item. In the brackets you will find the results from the first Delphi round.

	1 very unimportant	2	3	4	5	6	7 very important	cannot evaluate this item
4.a Information about the sources of funding (74% agreement on 6 and 7; Mean=6; S=1.3; n=91)	○	○	○	○	○	○	○	○
4.d Information about the project’s background (68% agreement on 6 and 7; Mean=5.9; S=1.1; n=90)	○	○	○	○	○	○	○	○
4.e Time of the Delphi study (55% agreement on 6 and 7; Mean=5.5; S=1.3; n=89)	○	○	○	○	○	○	○	○
4.g Information about the ethics vote (60% agreement on 6 and 7; Mean=5.5; S=1.6; n=82)	○	○	○	○	○	○	○	○

4.h Reference to additional information or materials about the project or Delphi study (e.g., online materials, Internet site) (53% agreement on 6 and 7; Mean=5.5; S=1.4; n=91)	◯	◯	◯	◯	◯	◯	◯	◯

Context

V5: If Delphi studies and their results are reported, how important do you consider the following aspects of the "Context - Section: Theory"?

Please respond with a “1” if you view an aspect as very unimportant or with a “7” if you view it as being very important. You can use the numbers in between to graduate the scale. You may also indicate that you cannot or do not wish to evaluate a particular item. In the brackets you will find the results from the first Delphi round.

	1 very unimportant	2	3	4	5	6	7 very important	cannot evaluate this item
5.a Positioning within the philosophy of science (e.g., realistic, positivist, constructivist) (24% agreement on 6 and 7; Mean=4.1; S=1.8; n=82)	◯	◯	◯	◯	◯	◯	◯	◯
5.b Identification of the research paradigm (in particular, qualitative or quantitative) (48% agreement on 6 and 7; Mean=5.1; S=1.6; n=89)	◯	◯	◯	◯	◯	◯	◯	◯
5.c Statement of presuppositions (e.g., regarding potentially contradictory topics) (48% agreement on 6 and 7; Mean=5.1; S=1.5; n=83)	◯	◯	◯	◯	◯	◯	◯	◯

Context

V6: If Delphi studies and their results are reported, how important do you consider the following aspects of the topic "Context - Sektion: Content"?

Please respond with a “1” if you view an aspect as very unimportant or with a “7” if you view it as being very important. You can use the numbers in between to graduate the scale. You may also indicate that you cannot or do not wish to evaluate a particular item. In the brackets you will find the results from the first Delphi round.

	1 very unimportant	2	3	4	5	6	7 very important	cannot evaluate this item
6.a Reflection on the relevance of the Delphi procedure as a topic, taking current research and the existing evidence base into account (52% agreement on 6 and 7; Mean=5.3; S=1.5; n=88)	◯	◯	◯	◯	◯	◯	◯	◯
6.b Reflection on the relevance of the Delphi procedure as a topic, taking social developments and innovations into account (e.g., the Covid-19 pandemic) (37% agreement on 6 and 7; Mean=6.5; S=1.5; n=91)	◯	◯	◯	◯	◯	◯	◯	◯
6.c Justification of the chosen method (Delphi procedure) to answer the research question (74% agreement on 6 and 7; Mean=6.1; S=1.1; n=91)	◯	◯	◯	◯	◯	◯	◯	◯

6.e Information about the combination of the Delphi procedure with other studies (e.g., mixed-methods design, triangulation) (67% agreement on 6 and 7; Mean=5.8; S= f. 1.3; n=87)	◯	◯	◯	◯	◯	◯	◯	◯

S4-6: How certain are you in responding to the topic "Context" of the Reporting Guideline?

Please respond with a “1” if you are extremely uncertain or with a “7” if you are absolutely certain. You can use the numbers in between to graduate the scale.

◯ 1 extremely uncertain
◯ 2
◯ 3
◯ 4
◯ 5
◯ 6
◯ 7 absolutely certain

O4-6: In the following you have the possibility to justify your answer and thus extend the list of arguments from the first Delphi round. If you do not have a new argument, you can skip the text field.

Please use the text box below. Please note: The text box is limited to 300 characters!

Open comments of the first Delphi round, sorted by topics

Set of items

- Some things are possible or nice to have, but not necessary.
- Reporting guidelines should focus on a minimal set, i.e. the essential items that must be reported by everyone. This approach differentiates the “nice to have” (many of the items in the last few questions) from the essential “must have”, such as the purpose of the Delphi.
- The topics highlighted are fundamental for a real advancement of research and a constructive brief between experts.
- There must be justification for a Delphi project when there are so many papers on the subject in the literature.
- Too much theoretical subtyping will damage the guideline in Medicine.
- Very important but may also lead to limitations of context

Importance depends on content/research process

- For me, Delphi studies are always qualitative, searching for arguments. Some questions above depends on the content. Transparency is key.
- The Delphi technique, as a hybrid research tool can be used as a stand-alone tool or in collaboration with other tools, in both quantitative and qualitative research. The objectives should be seen as seamless continuum from start to finish.
- A lot of this depends on the type of issues being discussed

Journal requirements

- Journals and readers do not like long, rambling background.
- I focused on publication in a journal rather than a report.

-

Method

V8: If Delphi studies and their results are reported, how important do you consider the following aspects of the "Method - Section: Delphi variations"?

Please respond with a “1” if you view an aspect as very unimportant or with a “7” if you view it as being very important. You can use the numbers in between to graduate the scale. You may also indicate that you cannot or do not wish to evaluate a particular item. In the brackets you will find the results from the first Delphi round.

	1 very unimportant	2	3	4	5	6	7 very important	cannot evaluate this item
8.a Identification of potential preliminary studies for the Delphi procedure (e.g., qualitative expert interviews, literature reviews) (64% agreement on 6 and 7; Mean=5.8; S=1.3; n=90)	○	○	○	○	○	○	○	○

DELPHISTAR
Delphi studies in health and social sciences – recommendations for a standardized reporting

Method

V9: If Delphi studies and their results are reported, how important do you consider the following aspects of the "Method - Section: Sample of experts"?

Please respond with a "1" if you view an aspect as very unimportant or with a "7" if you view it as being very important. You can use the numbers in between to graduate the scale. You may also indicate that you cannot or do not wish to evaluate a particular item. In the brackets you will find the results from the first Delphi round.

	1 very unimportant	2	3	4	5	6	7 very important	cannot evaluate this item
9.a Selection criteria for the experts (per round if there are different expert groups) (64% agreement on 6 and 7; Mean=6.5; S=0.9; n=91)	○	○	○	○	○	○	○	○
9.b Identification of the experts (64% agreement on 6 and 7; Mean=6; S=1.5; n=90)	○	○	○	○	○	○	○	○
9.c Information about recruiting and any subsequent recruiting of experts (74% agreement on 6 and 7; Mean=5.9; S=1.3; n=91)	○	○	○	○	○	○	○	○
9.e Information about how refusals and dropouts are handled (67% agreement on 6 and 7; Mean=5.8; S=1.5; n=91)	○	○	○	○	○	○	○	○

9.f Information about data protection regarding the experts (54% agreement on 6 and 7; Mean 5.3; S 1.7; n=91)	◯	◯	◯	◯	◯	◯	◯	◯
9.g Anonymity of the experts (48% agreement on 6 and 7; Mean=5; S=1.9; n=90)	◯	◯	◯	◯	◯	◯	◯	◯
9.h Information about the experts' sociodemographics per round (40% agreement on 6 and 7; Mean=4.8; S=1.8; n=90)	◯	◯	◯	◯	◯	◯	◯	◯
9.i Information about expert competency per round (55% agreement on 6 and 7; Mean=5.3; S=1.6; n=86)	◯	◯	◯	◯	◯	◯	◯	◯

Method

V10: If Delphi studies and their results are reported, how important do you consider the following aspects of the "Method - Section: Survey"?

Please respond with a “1” if you view an aspect as very unimportant or with a “7” if you view it as being very important. You can use the numbers in between to graduate the scale. You may also indicate that you cannot or do not wish to evaluate a particular item. In the brackets you will find the results from the first Delphi round.

	1 very unimportant	2	3	4	5	6	7 very important	cannot evaluate this item
10.a Elucidation of the content development for the questionnaire (2) (72% agreement on 6 and 7; Mean=6; S=1.2; n=88)	◯	◯	◯	◯	◯	◯	◯	◯
10.b Structure of the questionnaire (e.g., sections, organization) (58% agreement on 6 and 7; Mean=5.6; S=1.4; n=91)	◯	◯	◯	◯	◯	◯	◯	◯
10.c Number of items (open, closed, hybrid) (73% agreement on 6 and 7; Mean=5.4; S=1.5; n=91)	◯	◯	◯	◯	◯	◯	◯	◯
10.d Reference to additional integrated materials or information (e.g., info boxes) (51% agreement on 6 and 7; Mean=5.1; S=1.6; n=89)	◯	◯	◯	◯	◯	◯	◯	◯

10.e Information about and justification of the types of scales used (54% agreement on 6 and 7; Mean=5.5; S=1.4; n=91)	○	○	○	○	○	○	○	○
10.f Information about the graphic design of the questionnaire (e.g., use of figures) (28% agreement on 6 and 7; Mean=4.4; S=1.4; n=91)	○	○	○	○	○	○	○	○
10.g Information about the validity of the items/scales (54% agreement on 6 and 7; Mean=5.3; S=1.6; n=90)	○	○	○	○	○	○	○	○
10.h Information about the query regarding the experts' degree of certainty or competency (40% agreement on 6 and 7; Mean=5; S=1.6; n=87)	○	○	○	○	○	○	○	○
10.i Information about the pretest for the questionnaire (39% agreement on 6 and 7; Mean=4.9; S=1.6; n=91)	○	○	○	○	○	○	○	○
10.j Length of time to fill out the questionnaire per round (31% agreement on 6 and 7; Mean=4.6; S=1.7; n=91)	○	○	○	○	○	○	○	○
10.k Information about the software used for the survey (e.g., soscisurvey, e-delphi) (39% agreement on 6 and 7; Mean=4.8; S=1.6; n=91)	○	○	○	○	○	○	○	○

(2) Note: We use the term “questionnaire” for the survey instrument regardless of whether quantitative or qualitative items are integrated or weighted.

Method

V12: If Delphi studies and their results are reported, how important do you consider the following aspects of the "Method - Section: Feedback"?

Please respond with a “1” if you view an aspect as very unimportant or with a “7” if you view it as being very important. You can use the numbers in between to graduate the scale. You may also indicate that you cannot or do not wish to evaluate a particular item. In the brackets you will find the results from the first Delphi round.

	1 very unimportant	2	3	4	5	6	7 very important	cannot evaluate this item
12.b Information about the form of the feedback (e.g., statistical, graphical) (60% agreement on 6 and 7; Mean=5.7; S=1.4; n=90)	◯	◯	◯	◯	◯	◯	◯	◯
12.c Information about any differentiated feedback (e.g., according to expert group) (66% agreement on 6 and 7; Mean=5.9; S=1.4; n=91)	◯	◯	◯	◯	◯	◯	◯	◯

S7-12: How certain are you in responding to the topic "Method" of the Reporting Guideline?

Please respond with a “1” if you are extremely uncertain or with a “7” if you are absolutely certain. You can use the numbers in between to graduate the scale.

- ◯ 1 extremely uncertain
- ◯ 2
- ◯ 3
- ◯ 4
- ◯ 5
- ◯ 6
- ◯ 7 absolutely certain

O7-12: In the following you have the possibility to justify your answer and thus extend the list of arguments from the first Delphi round. If you do not have a new argument, you can skip the text field.

Please use the text box below. Please note: The text box is limited to 300 characters!

Open comments of the first Delphi round, sorted by topics

Experts

- More information on sample, e.g., possible conflicts of interest, what organizations/professional bodies they represent, funding sources/compensation for participating, number of patient panelists
- Expertise rapidly reaches a point of diminishing returns. Better hire as many of the cheapest experts as you can get than expensive experts.
- Because it is so hard to define and measure competence, the concept is not useful.
- Experts are key informants. In order to judge them it is crucial to know them, so

they cannot be anonymous. The whole validity of the method is dependent on their expertise.

Structure of the questionnaire

- Best to ensure that the persons understand what they are predicting through questionnaire structure.

Feedback/Supplemental material

- The outcomes from one round to the next are strategic for the clarity of the answers and for the self-assessment of the degree of competence by each expert.
- Feedback is very important to reach conclusions.
- An overlooked important point is the provision of supplemental material with each round. Reporting is less important when we have access to the actual survey tools.
- Information about any differentiated feedback (12c) is very important, if appropriate and used. But it is very unimportant otherwise.

Differentiation and importance of the items in a reporting guideline

- We need to figure out what is optional or when appropriate and then rate importance.
- Good practice in research design (with specific reference to Delphi methods) is what people need to do regardless of the method.
- Reporting guidelines should be a minimum set of essential items that all Delphi studies must report on - many of the suggested items are unduly burdensome and are better left to elaboration and explanation guidance.
- I would report some more information in the survey itself than in the reporting guideline. Some items I would include reworded.
- The roles of the experts, communication, disclosures of process are critical to the success of a Delphi. The research should ensure that there are no ambiguities, to validate the outcome of the exercise.

Data analysis and results

V13: If Delphi studies and their results are reported, how important do you consider the following aspects of the "Data analysis and results - Section: Data analysis"?

Please respond with a “1” if you view an aspect as very unimportant or with a “7” if you view it as being very important. You can use the numbers in between to graduate the scale. You may also indicate that you cannot or do not wish to evaluate a particular item. In the brackets you will find the results from the first Delphi round.

	1 very unimportant	2	3	4	5	6	7 very important	cannot evaluate this item
13.b. Information about the software used for analysis (e.g., SPSS, R, MAXQDA) (54% agreement on 6 and 7; Mean=5.3; S=1.5; n=91)	○	○	○	○	○	○	○	○

Data analysis and results

V14: If Delphi studies and their results are reported, how important do you consider the following aspects of the "Data analysis and results - Section: Delphi process"?

Please respond with a “1” if you view an aspect as very unimportant or with a “7” if you view it as being very important. You can use the numbers in between to graduate the scale. You may also indicate that you cannot or do not wish to evaluate a particular item. In the brackets you will find the results from the first Delphi round.

	1 very unimportant	2	3	4	5	6	7 very important	cannot evaluate this item
14.a Illustration of the Delphi process (e.g., in a flow chart) (57% agreement on 6 and 7; Mean=5.5; S=1.5; n=91)	◯	◯	◯	◯	◯	◯	◯	◯
14.b Information about special aspects during the Delphi process (e.g., deviations from the intended approach with justification) (73% agreement on 6 and 7; Mean=5.9; S=1.2; n=91)	◯	◯	◯	◯	◯	◯	◯	◯

S13-15: How certain are you in responding to the topic "Data analysis and results" of the Reporting Guideline?

Please respond with a “1” if you are extremely uncertain or with a “7” if you are absolutely certain. You can use the numbers in between to graduate the scale.

- ◯ 1 extremely uncertain
- ◯ 2
- ◯ 3
- ◯ 4
- ◯ 5
- ◯ 6
- ◯ 7 absolutely certain

O13-15: In the following you have the possibility to justify your answer and thus extend the list of arguments from the first Delphi round. If you do not have a new argument, you can skip the text field.

Please use the text box below. Please note: The text box is limited to 300 characters!

Open comments of the first Delphi round, sorted by topics

Feedback/ Communication

- … can be harmful. Often accuracy improves with repeated polling, e.g., "follow-the-leader" effects, but not feedback. Correlation between reduced variability and accuracy is hardly proven.
- …should include a clear communication of the methods of analysis, the presentation of progressive and final results to all participants.

Delphi variant-specific differences

- Policy Delphi and regular Delphi must be distinguished. They overlap to some

extent but have different goals.

Items in a reporting guideline

- Journals will have limits and disciplines will have expectations. 15a (Presentation of the results for each Delphi round and the final results) seems constraining.
- These items have to be reported in a Delphi study of excellence, but the structure has to be set up for it from the beginning.
- Surprised that there are so few items relating to the results. I suspect many users of the guideline would benefit from more clearly articulated recommendations on how they should report the results of the Delphi rounds.

General aspects

- Delphi study is the views of experts and this should be reported properly and in a transparent manner.
- One should not force answers in the online questionnaire in Delphi studies, because experts are sometimes uncertain about their answers.
- Some of the exceptional situations will always be problematic - and I would thus limit their scope.

-

Discussion and Dissemination

V16: If Delphi studies and their results are reported, how important do you consider the following aspects of the "Discussion and Dissemination - Section: Quality of findings"?

Please respond with a "1" if you view an aspect as very unimportant or with a "7" if you view it as being very important. You can use the numbers in between to graduate the scale. You may also indicate that you cannot or do not wish to evaluate a particular item. In the brackets you will find the results from the first Delphi round.

	1 very unimportant	2	3	4	5	6	7 very important	cannot evaluate this item
16.b Reliability of the results (e.g., when analyzing open-ended responses, intersubjectivity) (70% agreement on 6 and 7; Mean=5.8; S=1.4; n=87)	◯	◯	◯	◯	◯	◯	◯	◯
16.c Validation of the findings (e.g., communicative feedback for the respondents) (62% agreement on 6 and 7; Mean=5.7; S=1.4; n=87)	◯	◯	◯	◯	◯	◯	◯	◯

Discussion and Dissemination

V17: If Delphi studies and their results are reported, how important do you consider the following aspects of the "Discussion and Dissemination - Section: Dissemination"?

Please respond with a “1” if you view an aspect as very unimportant or with a “7” if you view it as being very important. You can use the numbers in between to graduate the scale. You may also indicate that you cannot or do not wish to evaluate a particular item. In the brackets you will find the results from the first Delphi round.

	1 very unimportant	2	3	4	5	6	7 very important	cannot evaluate this item
17.a Availability of the dataset (57% agreement on 6 and 7; Mean=5.3; S=1.7; n=91)	○	○	○	○	○	○	○	○
17.b Information about whether the results were made available to the experts (68% agreement on 6 and 7; Mean=5.7; S=1.6; n=90)	○	○	○	○	○	○	○	○
17.c Accessibility of the results for interested members of the public (50% agreement on 6 and 7; Mean=5.2; S=1.7; n=91)	○	○	○	○	○	○	○	○
17.d Information about further use of the results (47% agreement on 6 and 7; Mean=5.2; S=1.5; n=90)	○	○	○	○	○	○	○	○

S16-17: How certain are you in responding to the topic "Discussion and Dissemination" of the Reporting Guideline?

Please respond with a “1” if you are extremely uncertain or with a “7” if you are absolutely certain. You can use the numbers in between to graduate the scale.

- ◯ 1 extremely uncertain
- ◯ 2
- ◯ 3
- ◯ 4
- ◯ 5
- ◯ 6
- ◯ 7 absolutely certain

O16-17: In the following you have the possibility to justify your answer and thus extend the list of arguments from the first Delphi round. If you do not have a new argument, you can skip the text field.

Please use the text box below. Please note: The text box is limited to 300 characters!

Open comments of the first Delphi round, sorted by topics

Quality of findings

- Delphi questions/items are not measurement tools. They collect subjective opinions (which may change during rounds) and are dependent on the sample.
- Not clear how to assess validity and reliability.
- … depends on the participants and competence. A Delphi is still based on expert opinion if it is not based on evidence.

Public availability of the dataset

- … is handled differently depending on the disciplinary area

- … depends on the research objectives.
- … is limited by funding and ethics.

Discussion

- … should reflect how the research findings resonate with earlier research efforts and provide solutions to current research objectives.
- Representative qualitative quotes from each round help better understand the breadth of the discussion and show that far ranging views were considered.
- Process transparency and continuous communication of results are fundamental. It is not so essential to discuss explicit future advances

Dissemination

- Questions are irrelevant to Delphi method because they are addressed by the journal and data use.

-

Block III: Personal Questions

P1: Which discipline do you feel you belong to the most?

Please mark the one that matches most closely.

- ◯ Humanities
- ◯ Health science
- ◯ Natural science
- ◯ Engineering science
- ◯ Other, specifically:

  ____

P2: In which country are you regularly employed at present?

Please mark the country that matches most closely.

- ◯ Argentina
- ◯ Australia
- ◯ Belgium
- ◯ Brazil
- ◯ Chile
- ◯ China
- ◯ Denmark
- ◯ Germany
- ◯ England/UK
- ◯ Finland
- ◯ France
- ◯ Greece
- ◯ India
- ◯ Iran

- ◯ Ireland
- ◯ Israel
- ◯ Italy
- ◯ Japan
- ◯ Canada
- ◯ Colombia
- ◯ Korea
- ◯ Lebanon
- ◯ Malaysia
- ◯ Mexico
- ◯ New Zealand
- ◯ Netherlands
- ◯ Nigeria
- ◯ Norway
- ◯ Austria
- ◯ Palestine
- ◯ Poland
- ◯ Portugal
- ◯ Russia
- ◯ Sweden
- ◯ Switzerland
- ◯ Serbia
- ◯ Singapore
- ◯ Slovenia
- ◯ Spain
- ◯ South Africa
- ◯ South Korea
- ◯ Taiwan
- ◯ Thailand
- ◯ Tunisia

◯ Turkey

◯ Hungary

◯ USA

◯ Other, specifically:

____

____

P3: How many <u>Delphi studies</u> have you already participated in (as a leader, a consultant or person carrying out the study, not as a respondent)?

Please state the number. If you do not know the exact number, please estimate how many.

____

P4: How many <u>Delphi publications</u> (with or without peer review process) have you already participated in?

Please state the number. If you do not know the exact number, please estimate how many.

____

P5: In which year did you first encounter a Delphi procedure?

If you do not know the exact year, please estimate when.

____

P6: How well do you assess your ability to apply the different Delphi variations?

Please mark the one that matches most closely.

	1 absolutely no ability	2	3	4	5	6	7 excellent ability	I don't know
Classic Delphi	◯	◯	◯	◯	◯	◯	◯	◯
Real-time Delphi	◯	◯	◯	◯	◯	◯	◯	◯
Group Delphi	◯	◯	◯	◯	◯	◯	◯	◯
Policy Delphi	◯	◯	◯	◯	◯	◯	◯	◯
Argumentative Delphi	◯	◯	◯	◯	◯	◯	◯	◯
Deliberative Delphi	◯	◯	◯	◯	◯	◯	◯	◯
Fuzzy Delphi	◯	◯	◯	◯	◯	◯	◯	◯
Other Delphi variation, specifically: ____________________ -	◯	◯	◯	◯	◯	◯	◯	◯

P7: Which of the following profiles best describes your expertise on Delphi studies?

Please mark the one that matches most closely.

- ◯ Delphi beginner
- ◯ Delphi user
- ◯ Delphi expert

P8: How would you best describe your response behavior as you filled out the questionnaire?

Please mark the best description.

- ◯ Considered
- ◯ Intuitive
- ◯ Sometimes considered/sometimes intuitive
- ◯ I can't say

In which language did you answer the questionnaire?

Please select.

- ◯ English
- ◯ In another language.

You have reached the end of the questionnaire. By participating, you have helped us a lot in the development of a reporting guideline for Delphi studies.

Thank you very much for your support!

We will contact you again in about 6 weeks for the second Delphi round.

If you have any questions, please do not hesitate to contact marlen.niederberger@ph-gmuend.de.

You can now close this page.
