# Supplementary material for: Delphi studies in social and health sciences—Recommendations for an interdisciplinary standardized reporting (DELPHISTAR). Results of a Delphi study
Source: PLoS One. 2024 Aug 26;19(8):e0304651. doi: 10.1371/journal.pone.0304651 (PMC11346927; doi:10.1371/journal.pone.0304651)
Supplement: S2 File — (ZIP) [file pone.0304651.s002.zip › S2 File/DELPHISTAR_questionnaire_3.pdf]

DELPHISTAR

Delphi studies in health and social sciences – recommendations for a **sta**ndardized reporting

Delphi studies in social and health sciences – recommendations for an interdisciplinary standardized reporting (DELPHISTAR)

Questionnaire for the third Delphi round

From: Niederberger, M.; Spranger, J. Deckert, S.; Hirt, J.; Homberg, A.; Köberich, S.; Kuhn, R.: Rommel, A.; Sonnberger, M. and the DEWISS network. Delphi studies in social and health sciences – recommendations for an interdisciplinary standardized reporting (DELPHISTAR). Results of a Delphi study.

More information at OSF (https://osf.io/gc4jk) and DEWISS (https://delphi.ph-gmuend.de/)

Note

We wish to point out that the questionnaire was sent out online using Unipark survey software. It is impossible to retain the exact formatting of the online version when converting it into a PDF file, which is why there may be differences in appearance between the two. The PDF version was created via Unipark.

Furthermore, the publication by Niederberger et al. titled "Delphi studies in social and health sciences – recommendations for an interdisciplinary standardized reporting (DELPHISTAR). Results of a Delphi study" focuses only on the questionnaire items connected with developing the reporting guideline. In addition to these, there were at the beginning of the survey two other topic blocks that were rated by the experts. This was not part of the paper. The questions and data regarding these two other topic blocks are still included in the questionnaire and dataset.

If there are any questions, please contact:

Prof. Dr. Marlen Niederberger
E-mail: marlen.niederberger(at)ph-gmuend.de
Department of Research Methods in Health Promotion and Prevention
Institute for Health Sciences, University of Education Schwäbisch Gmünd,
Oberbettringer Strasse 200, 73525 Schwäbisch Gmünd, Germany

DELPHISTAR
Delphi studies in health and social sciences – recommendations for a standardized reporting

Note

Dear experts, we have formulated the questionnaire in English. It is possible that the questionnaire is automatically translated into your national language due to your browser settings. Unfortunately, this may result in translation errors. To ensure the correctness of the content, we ask you to switch off automatic translation via the browser.
In Google Chrome you can change this under Settings, Advanced, Languages.
In Firefox, you will find the setting under the "Translate Web Pages" add-on (if installed).
In Microsoft Edge you will find the selection under Settings, Languages or you will be directly shown a menu for selection where you can decline the translation.

Remarks on the evaluation of the second Delphi round

Questions that are in consensus according to our definition are no longer shown in this Delphi round. We used the following consensus definition:

- For items that should be included in the reporting guideline: Based on the seven-point rating scale at least 75% of the responses are for scale values 6 and 7 (agreement).
- For items that should **not** be included in the reporting guideline: Based on the seven-point rating scale less than 50% of the responses are for scale values 6 and 7 (agreement).

The **open-ended responses** were qualitatively evaluated using content analysis to clarify the wording of the items and add missing content.

Reformulated passages, compared to those of the previous round, are highlighted in color (blue).

Items that we define as consensus are no longer evaluated in this Delphi round, but are still listed if necessary to understand the questions that follow. You will also **see your individual response** from the first Delphi round for each item.

Statistical terms and abbreviations in the questionnaire

- **n**: number of cases
- **Mean**: „average“
- **Standard deviation (S)**: average deviation of the observed values from the mean value

DELPHISTAR
Delphi studies in health and social sciences – recommendations for a standardized reporting

Block I: Characteristics of a Delphi Procedure

This section involves identifying the important characteristics of Delphi studies. The reporting guideline will refer to this definition.

V1: In your opinion, how important or unimportant are the following characteristics for a Delphi study?

Different characteristics that define a Delphi procedure are discussed in the methods literature (literature references are at the end of this page). Please evaluate the importance of each characteristic for a Delphi study.

Please respond with a "1" if you consider the characteristic to be very unimportant or with a "7" if you consider it to be very important. You can use the numbers in between to graduate the scale. You may also indicate that you cannot or do not wish to evaluate a particular item.
In the brackets you will find the results from the second Delphi round.

	1 very unimportant	2	3	4	5	6	7 very important	cannot evaluate this item
1.2 Structured communication process that involves a group of people with the relevant expertise (68% agreement on importance (6 and 7); Mean=5.9; S=1.3; n=69)	◯	◯	◯	◯	◯	◯	◯	◯

1.3 Option to remain anonymous to the other participating experts and to give the individual answer in a safe space (64% agreement on importance (6 and 7); Mean=5.5; S=1.6; n=69)	○	○	○	○	○	○	○	○
1.6 Basis is a quantitative questionnaire with the possibility to contribute or supplement arguments for the respective position (70% agreement on importance (6 and 7); Mean=5.8; S=1.2; n=69)	○	○	○	○	○	○	○	○
1.7 All answers, quantitative and qualitative, are systematically analyzed (quantitative: e.g. descriptive statistics, qualitative: e.g. thematic analysis) (57% agreement on importance (6 and 7); Mean=5.4; S=1.6; n=69)	○	○	○	○	○	○	○	○

S1: We would now be interested in your personal assessment of your certainty of judgement. How certain are you in responding to this question?

Please respond with a “1” if you are extremely uncertain or with a “7” if you are absolutely certain. You can use the numbers in between to graduate the scale.

- ◯ 1 extremely uncertain
- ◯ 2
- ◯ 3
- ◯ 4
- ◯ 5
- ◯ 6
- ◯ 7 absolutely certain

Literature

- Linstone, H.A. M. Turoff, M (1975): The delphi method, Addison-Wesley, MA.
- Niederberger, M.; Spranger, J. (2020): Delphi technique in health sciences: A Map, Front. Public Health 8, 1–10.
- Rowe, G.; Wright, G.; Bolger, F. (1991): Delphi, A reevaluation of research and theory. Technological Forecasting and Social Change (39/3), S.235-251.
- Turoff, M.; Linstone, H.A. (2002 Eds.): The Delphi Method: Techniques and Applications, Addison-Wesley, Boston.
- Von der Gracht, H.A. (2012): Consensus measurement in Delphi studies. Review and implications for future quality assurance. In: Technological Forecasting and Social Change, 79, S. 1525-1536.

V2: Although reporting guidelines have proven effective in the health sciences for establishing standards and quality control for academic publications, there are no reporting guidelines for Delphi studies yet that are recognized by different disciplines and that consider the different distinctive ways in which they may be conducted.

a) In your opinion, how likely is the development of an expert-agreed reporting guideline for different Delphi variants (e.g., classical Delphi, real-time Delphi)?

Please respond with a “1” if you view the development of a reporting guideline to be very unlikely or with a “7” if you view this as being very likely. You can use the numbers in between to graduate the scale. You may also indicate that you cannot or do not wish to evaluate this item.

- ◯ 1 very unlikely
- ◯ 2
- ◯ 3
- ◯ 4
- ◯ 5
- ◯ 6
- ◯ 7 very likely
- ◯ cannot evaluate this item

b) In your opinion, how likely is the development of an expert-agreed reporting guideline on Delphi studies which explicitly applies to the social and health sciences?

Please respond with a “1” if you view the development of a reporting guideline to be very unlikely or with a “7” if you view this as being very likely. You can use the numbers in between to graduate the scale. You may also indicate that you cannot or do not wish to evaluate this item.

- ◯ 1 very unlikely
- ◯ 2
- ◯ 3
- ◯ 4
- ◯ 5
- ◯ 6
- ◯ 7 very likely
- ◯ cannot evaluate this item

S2: How certain are you in responding to these questions?

Please respond with a “1” if you are extremely uncertain or with a “7” if you are absolutely certain. You can use the numbers in between to graduate the scale.

- ◯ 1 extremely uncertain
- ◯ 2
- ◯ 3
- ◯ 4
- ◯ 5
- ◯ 6
- ◯ 7 absolutely certain

DELPHISTAR
Delphi studies in health and social sciences – recommendations for a standardized reporting

Block II: Reporting Guideline

Context

V4: If Delphi studies and their results are reported, how important do you consider the following aspects of the "Context - Section: Formal"?

Please respond with a “1” if you view an aspect as very unimportant or with a “7” if you view it as being very important. You can use the numbers in between to graduate the scale. You may also indicate that you cannot or do not wish to evaluate a particular item. In the brackets you will find the results from the second Delphi round.

	1 very unimportant	2	3	4	5	6	7 very important	cannot evaluate this item
4.e Time period in which the Delphi study was conducted (66% agreement on 6 and 7; Mean=5.7; S=1.3; n=67)	◯	◯	◯	◯	◯	◯	◯	◯
4.g Information on the ethics vote should be provided. This also includes indicating if no vote was required by the responsible ethics committee (62% agreement on 6 and 7; Mean=5.5; S=1.5; n=61)	◯	◯	◯	◯	◯	◯	◯	◯

4.h Reference to additional information or materials about the project or Delphi study (e.g., online questionnaire, website on the project background) (52% agreement on 6 and 7; Mean=5.4; S=1.5; n=69)	◯	◯	◯	◯	◯	◯	◯	◯

Context

V5: If Delphi studies and their results are reported, how important do you consider the following aspects of the "Context - Section: Theory"?

Please respond with a “1” if you view an aspect as very unimportant or with a “7” if you view it as being very important. You can use the numbers in between to graduate the scale. You may also indicate that you cannot or do not wish to evaluate a particular item. In the brackets you will find the results from the second Delphi round.

	1 very unimportant	2	3	4	5	6	7 very important	cannot evaluate this item
5.b Identification of the research paradigm (qualitative or quantitative or Mixed Methods) (52% agreement on 6 and 7; Mean=5.2; S=1.6; n=68)	○	○	○	○	○	○	○	○

V6: If Delphi studies and their results are reported, how important do you consider the following aspects of the topic "Context - Sektion: Content"?

Please respond with a “1” if you view an aspect as very unimportant or with a “7” if you view it as being very important. You can use the numbers in between to graduate the scale. You may also indicate that you cannot or do not wish to evaluate a particular item. In the brackets you will find the results from the second Delphi round.

	1 very unimportant	2	3	4	5	6	7 very important	cannot evaluate this item
6.a Highlight why the Delphi study is relevant (e.g., due to research gaps or practical relevance to avoid "research waste") (61% agreement on 6 and 7; Mean=5.5; S=1.6; n=66)	○	○	○	○	○	○	○	○
6.e Information if the Delphi study is combined with another study (e.g., systematic review to develop the questionnaire, focus group with patients to discuss the Delphi results) (71% agreement on 6 and 7; Mean=5.8; S=1.3; n=68)	○	○	○	○	○	○	○	○

S4-6: How certain are you in responding to the topic "Context" of the Reporting Guideline?

Please respond with a “1” if you are extremely uncertain or with a “7” if you are absolutely certain. You can use the numbers in between to graduate the scale.

- ◯ 1 extremely uncertain
- ◯ 2
- ◯ 3
- ◯ 4
- ◯ 5
- ◯ 6
- ◯ 7 absolutely certain

Method

V9: If Delphi studies and their results are reported, how important do you consider the following aspects of the "Method - Section: Sample of experts"?

Please respond with a “1” if you view an aspect as very unimportant or with a “7” if you view it as being very important. You can use the numbers in between to graduate the scale. You may also indicate that you cannot or do not wish to evaluate a particular item. In the brackets you will find the results from the second Delphi round.

	1 very unimportant	2	3	4	5	6	7 very important	cannot evaluate this item
9.e Information about how refusals and dropouts are handled (e.g., number of reminders, non-response analyses) (67% agreement on 6 and 7; Mean=5.8; S=1.4; n=69)	○	○	○	○	○	○	○	○
9.i Information about expert competency (e.g., via professional experience, institutional affiliation, expertise in relevant fields/disciplines, conflict of interests) (56% agreement on 6 and 7; Mean=5.3; S=1.6; n=64)	○	○	○	○	○	○	○	○

Method

V10: If Delphi studies and their results are reported, how important do you consider the following aspects of the "Method - Section: Survey"?

Please respond with a “1” if you view an aspect as very unimportant or with a “7” if you view it as being very important. You can use the numbers in between to graduate the scale. You may also indicate that you cannot or do not wish to evaluate a particular item. In the brackets you will find the results from the second Delphi round.

	1 very unimportant	2	3	4	5	6	7 very important	cannot evaluate this item
10.b Description of the questionnaire (content and structure) (71% agreement on 6 and 7; Mean=5.8; S=1.3; n=69)	○	○	○	○	○	○	○	○
10.c Number of questions (open, closed, hybrid) (61% agreement on 6 and 7; Mean=5.6; S=1.5; n=69)	○	○	○	○	○	○	○	○
10.d Reference to additional integrated materials or information (e.g., info boxes illustrating the current knowledge about the theme focused on) (55% agreement on 6 and 7; Mean=5.2; S=1.5; n=67)	○	○	○	○	○	○	○	○

10.e Information about and justification of the types of scales used (e.g., nominal scales, rating or ranking scales) (58% agreement on 6 and 7; Mean=5.6; S=1.4; n=69)	○	○	○	○	○	○	○	○
10.g Information about the validity of the items/scales (e.g., information on the piloting of the questionnaire or the evaluation of validity) (53% agreement on 6 and 7; Mean=5.0; S=1.6; n=65)	○	○	○	○	○	○	○	○

Note: We use the term “questionnaire” for the survey instrument regardless of whether quantitative or qualitative items are integrated or weighted.

Method

V12: If Delphi studies and their results are reported, how important do you consider the following aspects of the "Method - Section: Feedback"?

Please respond with a “1” if you view an aspect as very unimportant or with a “7” if you view it as being very important. You can use the numbers in between to graduate the scale. You may also indicate that you cannot or do not wish to evaluate a particular item. In the brackets you will find the results from the second Delphi round.

	1 very unimportant	2	3	4	5	6	7 very important	cannot evaluate this item
12.b Information on how the results of the previous Delphi round were fed back to the experts surveyed (e.g., via frequencies, mean values, measures of dispersion, listing of comments) (63% agreement on 6 and 7; Mean=5.7; S=1.3; n=68)	○	○	○	○	○	○	○	○
12.c Information on whether feedback was differentiated by specific groups (e.g., by field of expertise, institutional affiliation) (73% agreement on 6 and 7; Mean=6.0; S=1.2; n=66)	○	○	○	○	○	○	○	○

S7-12: How certain are you in responding to the topic "Method" of the Reporting Guideline?

Please respond with a “1” if you are extremely uncertain or with a “7” if you are absolutely certain. You can use the numbers in between to graduate the scale.

- ◯ 1 extremely uncertain
- ◯ 2
- ◯ 3
- ◯ 4
- ◯ 5
- ◯ 6
- ◯ 7 absolutely certain

Data Analysis and Results

V13: If Delphi studies and their results are reported, how important do you consider the following aspects of the "Data Analysis and Results - Section: Data analysis"?

Please respond with a “1” if you view an aspect as very unimportant or with a “7” if you view it as being very important. You can use the numbers in between to graduate the scale. You may also indicate that you cannot or do not wish to evaluate a particular item. In the brackets you will find the results from the second Delphi round.

	1 very unimportant	2	3	4	5	6	7 very important	cannot evaluate this item
13.b. Information about the software used for analysis (e.g., SPSS, R, MAXQDA) (55% agreement on 6 and 7; Mean=5.4; S=1.5; n=69)	○	○	○	○	○	○	○	○

V14: If Delphi studies and their results are reported, how important do you consider the following aspects of the "Data Analysis and Results - Section: Delphi process"?

Please respond with a “1” if you view an aspect as very unimportant or with a “7” if you view it as being very important. You can use the numbers in between to graduate the scale. You may also indicate that you cannot or do not wish to evaluate a particular item. In the brackets you will find the results from the second Delphi round.

	1 very unimportant	2	3	4	5	6	7 very important	cannot evaluate this item
14.a Illustration of the Delphi process (e.g., in a flow chart) (70% agreement on 6 and 7; Mean=5.7; S=1.5; n=69)	◯	◯	◯	◯	◯	◯	◯	◯

S13-15: How certain are you in responding to the topic "Data Analysis and Results" of the Reporting Guideline?

Please respond with a “1” if you are extremely uncertain or with a “7” if you are absolutely certain. You can use the numbers in between to graduate the scale.

◯ 1 extremely uncertain
◯ 2
◯ 3
◯ 4
◯ 5
◯ 6
◯ 7 absolutely certain

Discussion and Dissemination

V16: If Delphi studies and their results are reported, how important do you consider the following aspects of the "Discussion and Dissemination - Section: Quality of findings"?

Please respond with a “1” if you view an aspect as very unimportant or with a “7” if you view it as being very important. You can use the numbers in between to graduate the scale. You may also indicate that you cannot or do not wish to evaluate a particular item. In the brackets you will find the results from the second Delphi round.

	1 very unimportant	2	3	4	5	6	7 very important	cannot evaluate this item
16.New Item: Highlighting the findings from the Delphi study	◯	◯	◯	◯	◯	◯	◯	◯
16.b Reliability of the results (e.g., how many people analyzed the qualitative responses) (68% agreement on 6 and 7; Mean=5.8; S=1.4; n=66)	◯	◯	◯	◯	◯	◯	◯	◯
16.c External validity of the findings (66% agreement on 6 and 7; Mean=5.7; S=1.3; n=64)	◯	◯	◯	◯	◯	◯	◯	◯

Discussion and Dissemination

V17: If Delphi studies and their results are reported, how important do you consider the following aspects of the "Discussion and Dissemination - Section: Dissemination"?

Please respond with a “1” if you view an aspect as very unimportant or with a “7” if you view it as being very important. You can use the numbers in between to graduate the scale. You may also indicate that you cannot or do not wish to evaluate a particular item. In the brackets you will find the results from the second Delphi round.

	1 very unimportant	2	3	4	5	6	7 very important	cannot evaluate this item
17.a Availability of the dataset (61% agreement on 6 and 7; Mean=5.3; S=1.7; n=69)	◯	◯	◯	◯	◯	◯	◯	◯

S16-17: How certain are you in responding to the topic "Discussion and Dissemination" of the Reporting Guideline?

Please respond with a “1” if you are extremely uncertain or with a “7” if you are absolutely certain. You can use the numbers in between to graduate the scale.

◯ 1 extremely uncertain
◯ 2
◯ 3
◯ 4
◯ 5
◯ 6
◯ 7 absolutely certain

Block III: Personal Questions

P1: Which discipline do you feel you belong to the most?

Please mark the one that matches most closely.

- ◯ Humanities
- ◯ Health science
- ◯ Natural science
- ◯ Engineering science
- ◯ Other, specifically:

  ____

P2: In which country are you regularly employed at present?

Please mark the country that matches most closely.

- ◯ Argentina
- ◯ Australia
- ◯ Austria
- ◯ Belgium
- ◯ Brazil
- ◯ Canada
- ◯ Chile
- ◯ China
- ◯ Colombia
- ◯ Denmark
- ◯ England/UK
- ◯ Finland
- ◯ France
- ◯ Germany

- ◯ Greece
- ◯ Hungary
- ◯ India
- ◯ Iran
- ◯ Ireland
- ◯ Israel
- ◯ Italy
- ◯ Japan
- ◯ Korea
- ◯ Lebanon
- ◯ Malaysia
- ◯ Mexico
- ◯ New Zealand
- ◯ Netherlands
- ◯ Nigeria
- ◯ Norway
- ◯ Palestine
- ◯ Poland
- ◯ Portugal
- ◯ Russia
- ◯ Serbia
- ◯ Singapore
- ◯ Slovenia
- ◯ South Africa
- ◯ South Korea
- ◯ Spain
- ◯ Sweden
- ◯ Switzerland
- ◯ Taiwan
- ◯ Thailand

- ◯ Tunisia
- ◯ Turkey
- ◯ USA
- ◯ Other, specifically: ______________________

P4: How many Delphi publications (with or without peer review process) have you already participated in?

Please state the number. If you do not know the exact number, please estimate how many.

____

P5: In which year did you first encounter a Delphi procedure?

If you do not know the exact year, please estimate when.

____

P6: How well do you assess your ability to apply the different Delphi variations?

Please mark the one that matches most closely.

	1 absolutely no ability	2	3	4	5	6	7 excellent ability	I don’t know
Classic Delphi	◯	◯	◯	◯	◯	◯	◯	◯
Real-time Delphi	◯	◯	◯	◯	◯	◯	◯	◯
Group Delphi	◯	◯	◯	◯	◯	◯	◯	◯
Policy Delphi	◯	◯	◯	◯	◯	◯	◯	◯
Argumentative Delphi	◯	◯	◯	◯	◯	◯	◯	◯
Deliberative Delphi	◯	◯	◯	◯	◯	◯	◯	◯
Fuzzy Delphi	◯	◯	◯	◯	◯	◯	◯	◯
Other Delphi variation, specifically: ______________________ -	◯	◯	◯	◯	◯	◯	◯	◯

P7: Which of the following profiles best describes your expertise on Delphi studies?

Please mark the one that matches most closely.

◯ Delphi beginner

◯ Delphi user

◯ Delphi expert

P8: How would you best describe your response behavior as you filled out the questionnaire?

Please mark the best description.

- ◯ Considered
- ◯ Intuitive
- ◯ Sometimes considered/sometimes intuitive
- ◯ I can't say

In which language did you answer the questionnaire?

Please select.

◯ English

◯ In another language.

Anything else you want to share with us?

Please write your comments on the survey in the text box below or click "CONTINUE".

-

Acknowledgement

Please select “Yes, I would like to be acknowledged” in the box below if you would like to be acknowledged in the published guidelines. We would also list you as a collaborator on the published reporting guidelines if the target journal provides this feature.

◯ Yes, I would like to be acknowledged

◯ No, please maintain my anonymity

If you select yes to the question above, please enter how you would like both, your name and affiliation, to be presented.

You have reached the end of the questionnaire. By participating, you have helped us a lot in the development of a reporting guideline for Delphi studies.

Thank you very much for your support!

We will contact you again in about 6 weeks for the second Delphi round.

If you have any questions, please do not hesitate to contact marlen.niederberger@ph-gmuend.de.

You can now close this page.
