# Supplementary material for: Delphi studies in social and health sciences—Recommendations for an interdisciplinary standardized reporting (DELPHISTAR). Results of a Delphi study
Source: PLoS One. 2024 Aug 26;19(8):e0304651. doi: 10.1371/journal.pone.0304651 (PMC11346927; doi:10.1371/journal.pone.0304651)
Supplement: Attachment — Submitted filename: Response to Reviewers_final ENGL.docx [file pone.0304651.s003.docx]

**Article: Delphi studies in social and health sciences – recommenda-tions for an interdisciplinary standardized reporting (DEL-PHISTAR). Results of a Delphi study.**

Dear editor,

Dear Dr. Felicity Hasson,

Dear Professor Jon Landeta,

Thank you for your valuable insights and comments on our manuscript, "Delphi studies in social and health sciences – recommendations for an interdisciplinary standardized reporting (DELPHISTAR). Results of a Delphi study." We have given your constructive feedback careful consideration and used it to improve the quality and clarity of our paper. The comments regarding the detailed description of the methodological approach have improved the transparency and understandability of our approach. Above all, reflecting on the study's limitations and the discussion section, we found your remarks to be a valuable way to expand on how to interpret the study results.

We very much appreciate the time and effort that you have spent evaluating our manuscript, and we are happy to send you now the revised version.

With kind regards,

Prof. Dr. Marlen Niederberger on behalf of the authors

| No. | Editor Comments | Author Responses |
| --- | --- | --- |
| 1 | 1. Please ensure that your manuscript meets PLOS ONE's style requirements, including those for file naming. The PLOS ONE style templates can be found at  <https://journals.plos.org/plosone/s/file?id=wjVg/PLOSOne_formatting_sample_main_body.pdf> and  <https://journals.plos.org/plosone/s/file?id=ba62/PLOSOne_formatting_sample_title_authors_affiliations.pdf> | Thank you for this information. We checked the formal requirements and have followed them. |
| 2 | 2. Did you know that depositing data in a repository is associated with up to a 25% citation advantage (https://doi.org/10.1371/journal.pone.0230416)? If you’ve not already done so, consider depositing your raw data in a repository to ensure your work is read, appreciated and cited by the largest possible audience. You’ll also earn an Accessible Data icon on your published paper if you deposit your data in any participating repository (https://plos.org/open-science/open-data/#accessible-data). | The team of authors have decided to make the data available by publishing them in the form of a supplement to the manuscript. |
| 3 | 3. Please provide additional details regarding participant consent. In the ethics statement in the Methods and online submission information, please ensure that you have specified (1) whether consent was informed and (2) what type you obtained (for instance, written or verbal, and if verbal, how it was documented and witnessed). | We have added information to make this complete. Thank you for pointing this out. |
| 4 | 4. Thank you for stating the following financial disclosure: "all authors Deutsche Forschungsgemeinschaft (DFG) - Projektnummer 429572724 Network promotion".  Please state what role the funders took in the study. If the funders had no role, please state: "The funders had no role in study design, data collection and analysis, decision to publish, or preparation of the manuscript."  If this statement is not correct you must amend it as needed.  Please include this amended Role of Funder statement in your cover letter; we will change the online submission form on your behalf. | We have specified the role of the funder. |
| 5 | 5. In the online submission form, you indicated that "The data underlying the results presented in the study are available from Prof. dr. Marlen Niederberger (marlen.niederberger@ph-gmuend.de)".  All PLOS journals now require all data underlying the findings described in their manuscript to be freely available to other researchers, either 1. In a public repository, 2. Within the manuscript itself, or 3. Uploaded as supplementary information.  This policy applies to all data except where public deposition would breach compliance with the protocol approved by your research ethics board. If your data cannot be made publicly available for ethical or legal reasons (e.g., public availability would compromise patient privacy), please explain your reasons on resubmission and your exemption request will be escalated for approval. | We will make the data available in the form of supplementary information. |
| 6 | MAJOR COMMENTS  The study has developed an e-Delphi, which was also described as a classic Delphi. The authors should describe better what is an e-Delphi, and rethink whether the study is a classic Delphi given the features that follow. | Thank you for making us aware of this. We have provided a more detailed explanation. It is possible that the term "e-Delphi" was misleading. We now refer to it as an "online Delphi." According to our understanding, a classic Delphi can also be conducted online. |
| 7 | There is an adoption of features in the Delphi study that raise issues – usually the items do not change in sequential rounds, but the authors describe “The initial questionnaire was revised based on the results of the first Delphi round, meaning that consented items were removed and the remaining items were reworded as necessary”. However rewording of items in sequential rounds raises issues for interpretation, comparability and analyses which are not reflected upon in the manuscript. Literature should frame why the procedure is acceptable, and I am not sure whether this is a classical Delphi, or its links to an enchained Delphi. | We have justified the rewording of items and cited similar approaches in other Delphi studies. |
| 8 | It would be important to understand whether participants were informed about the selected majority rules prior to the Delphi. | This is an important point which we have expanded on in more detail. |
| 9 | The authors need to explain how Table 1 was generated. | We have added a brief description and hope that this contributes toward making the table understandable. |
| 10 | The authors need to explain the rationale and roots for using the selected importance scale. | We have followed your advice and have justified the selection of the scale. |
| 11 | The discussion on whether the proposed guideline is useful for distinct types of Delphi process types/variants needs to be extended and deepened (classical Delphi, policy Delphi, etc). | We have explained where differences lie between the Delphi variants. |
| 12 | I do not understand the appropriateness of using the sentence “None of the experts faced any repercussions for deciding not to participate or for dropping out”. | We have deleted this sentence. |
| 13 | As the manuscript claims that a key output is a complete guideline list, I wonder whether it be presented or summarized within the manuscript. | The reporting guideline has been included in the supplement. |
| 14 | The manuscript does not discuss future research (following the work done), as well as should go deeper in the study limitations. | We noted at the end of the discussion section that we want to test the reporting guideline in a further step so as to be able to make a statement regarding feasibility.  We have described the study limitations in more depth and detail. With their valuable feedback, the reviewers have helped us reflect on this part of the paper and expand it with new perspectives. |
| 15 | The authors should provide more detailed information on the search protocol used to get the two Zotero databases. | Thank you for this comment. The creation of the databases is described in more detail in the cited publication (Spranger et al. 2022). Nevertheless, we have described more precisely which criteria were applied to the search. |
| 16 | MINOR COMMENTS  I missed an explicit statement of the objectives before the methods section. | We have made the study objectives more explicit, namely prior to the section on the background of the study. |
| 17 | The authors should comment on what could be done to avoid “some of the emails were blocked”. | We have found a publication with tips on how to improve the delivery of emails. We have made reference to these tips and cited the publication. |
|  | Reviewer 1 (Dr. Felicity Hasson) |  |
| 1 | Thank you for allowing me the opportunity to review this paper it was a delight to read. The study adds to the evidence based relating to the reporting of Delphi Studies. Whilst such guidelines do exist, this study builds upon this evidence and recent application examples to formulate a 3-round modified Delphi. The authors should be commended for this in-depth approach adopted. See below some minor reflections/ suggestions: | Dear Dr. Hasson,  Thank you for reviewing our manuscript and sharing your expertise with us in the form of detailed and well-founded feedback. The comments were very helpful to us and have improved the quality of the manuscript. |
| 2 | As an observation, the rationale underpinning the use of guidelines, could be brought stronger to the fore for example, to address methodological misunderstandings, enhance the rigour and quality. Moreover, this study proposes addressing the need for nonspecific areas but generically across health and social are sciences needs to be brought to the fore in the background, as well as consideration for why this wide area needs this evidence (i.e., most utilised within this field?). | We have made this more explicit and in regard to topical areas we cite the study by Khodyakov et al. (2023), which applies bibliometric analysis to investigate disciplinary trends in Delphi studies. Thank you for this comment. |
| 3 | The low uptake of the Delphi within this study from the total sample targeted also needs to be addressed and may be related to the inclusion criteria required to read and speak English, hence limiting participation. | This is an important aspect that, up to now, has not been sufficiently addressed in our manuscript. This was also noticed by the editor, and we have expanded on this aspect in more depth. |
| 4 | There is a sense from the discussion that this should be applied but dependent upon journals acceptability. However, would further expert panel consultation not also be beneficial, prior to implementation? Moreover, how authors apply this may also need to be developed. We assume the existing frameworks are appropriate but some blue sky thinking of how to move these forwards are rarely discussed. | We have reformulated and specified how the reporting guideline should be disseminated. We want to realize the dissemination of the reporting guideline through promotion via a website and short videos for social media posts and by contacting major journals in the field of Delphi studies and Delphi practitioners. |
|  | Reviewer 2 (Prof. Jon Landeta) |  |
| 1 | The article presented is an interesting and necessary contribution to the study and improvement of the Delphi methodology. It is the result of a systematic and well-oriented research process, which has concluded with a Delphi study to propose the most important items, agreed with the scientific community that usually uses this technique, which should be included in the reporting of a Delphi study. This is the next logical and necessary step after the previous study carried out by the Niederberger and Spranger team (2020), which had conducted a systematic review of the methodological work published to date on the Delphi method and on the elements that the various authors recommended to include in the reporting of results.  As could not be otherwise, in this study the method has been used with rigor, the group of experts consulted has been large and justified, the number of responses obtained is sufficient, the results are valuable and the reporting is very well done.  Nevertheless, we would like to make some comments or suggestions, in the hope that the authors will assess their applicability to the improvement of this work. | Dear Professor Landeta,  Thank you for your positive response to our manuscript and your helpful feedback. We are happy to hear that you have a high estimation of our study on Delphi methodology. Your comments and insights are important to us, and we have given them careful consideration and made changes to our manuscript based on them. Our responses to the separate comments are listed below. |
| 2 | - Selection of experts. The expert selection process is perfectly justified and defined in the report. However, the decision criteria selected may, in our opinion, have excluded potential quality contributions. In other words, all participants are experts, but perhaps not all genuine experts were able to participate. The criterion of limiting the search to authors who have published between 2016 and 2021 may have left out relevant authors. In the list of published experts I miss the main methodologists of this technique, alive, according to the number of citations that their works have accumulated: Rowe, Wright, Okoli, Pawlowski, Adler, Ziglio, Skulmoski, Krann, Landeta, Von der Gracht, Gordon, Pease, Tapio, Turoff, Hasson... They may have been invited and declined to participate, or they did not want to publicize their names, but the reality is that none of the most cited appears. Moreover, the profile of the experts consulted corresponds mainly to Health Sciences, and very little to Social Sciences. It is not a question of repeating the study, but of indicating more clearly this deficiency in the final limitations. | This is an important criticism that we have addressed in the description of the study limitations. We also wrote to many of the methods experts but are unable to give more details for reasons of data privacy. |
| 3 | - In order to accept the inclusion of an item in the final list of items to be included in a report, the criterion was that more than 75% of the experts who responded rated its importance with 6 or 7 points (out of 7). This is a criterion, but I believe it is too restrictive and leads to the loss of valuable information. It is true that in the tables the % of consensus on each item is maintained, but the presentation of results simplifies the analysis with a Yes or No item. | This would have been a way to determine consensus. Perhaps even more advantageously in some regards due to the ability to better differentiate, as explained by you. However, because we shared the definition of consensus with the surveyed experts and even defined it ahead of time, we will be retaining it. Also, it became clear from the experts' open responses that they feel it is important to agree on a minimum of essential criteria.  That said, we receive this feedback very gladly in terms of future studies. Moreover, we have included a self-criticism in the limitations. |
|  | In my opinion, all the items included (65) have their importance and their reason to be in a final report, even some more could have been. Therefore, the results should be, at least, classified in three categories:  a. Items of highly consensual, necessary and recommended inclusion (e.g., above 75% consensus).  b. Items of desirable and generally necessary inclusion (e.g., between 50% and 75%).  c. Possible inclusion items, depending on the study and study objectives (less than 50%).  Or something similar. |  |
| 4 | - The contribution of the work is valuable and necessary, but at the end of the reading a slight disappointment remains, because a study carried out with such rigor and with the participation of so many experts is limited to providing a list of items to be included in a reporting hierarchy according to the degree of consensus they have reached. We receive no information on the reasoning that these people have used to support their position in favor, or not, of considering each item as very important. I would ask the authors to provide in an auxiliary document the main arguments for and against the inclusion of each item in the final list, gathered from the qualitative contributions of the experts. | In the results section, we referred to the open comments if we felt it was relevant. However, we can understand that interested readers may want more detailed information. As a way to enable this, the author team has decided to make the experts' arguments available as a supplement. |
| 5 | - There are two final items that have obtained a high consensus Reliability of the results (80%) and External validity of the findings (74%) that could have been worked on more in this study, providing indicators of the quality of the work based on judgments external to the authors. For example, including a survey of the participating experts in which they are asked about the rigor with which the study was conducted, their confidence in the results, their satisfaction with the participation... A recent publication on this subject is Landeta and Lertxundi (2023). Quality indicators for Delphi studies. Futures & Foresight Science, e172. | We have added this point to the discussion and cite the publication mentioned, which is very relevant here and which we read with great interest. |
| 6 | In summary, it is an interesting and necessary article, which could be improved by considering the comments made on the limitations of its expert participants, the classification of the items analyzed, the additional qualitative information that could be provided and the inclusion of some indicator of the quality of the study external to the authors who carried it out. | Thank you for your thorough review of our manuscript and your well-thought-out comments. They have helped us revise the manuscript, enabling a more extensive and robust presentation of our study. We have taken all of the points into consideration while making changes. This letter contains our explanations on how we did this. |
