# Supplementary material for: Delphi studies in social and health sciences—Recommendations for an interdisciplinary standardized reporting (DELPHISTAR). Results of a Delphi study
Source: PLoS One. 2024 Aug 26;19(8):e0304651. doi: 10.1371/journal.pone.0304651 (PMC11346927; doi:10.1371/journal.pone.0304651)
Supplement: Attachment — Submitted filename: Response to Reviewers_2 ENGL.docx [file pone.0304651.s004.docx]

Dear editor,

Dear reviewer,

Thank you for your prompt response and positive evaluation of our paper "Delphi studies in social and health sciences – recommendations for an interdisciplinary standardized reporting (DELPHISTAR). Results of a Delphi study". We have re-added the missing item to the list of Delphi characteristics and are grateful to you for drawing our attention to it. Furthermore, we have minimally changed the phrasing in several sentences. All of the changes are highlighted in color.

Overall, we want to thank you for the helpful information and respect shown during the entire review process. We are happy to have the paper published soon.

With kind regards,

Prof. Dr. Marlen Niederberger on behalf of the authors

| No. | Review Comments to the Author | Author Responses |
| --- | --- | --- |
| 1 | Please use the space provided to explain your answers to the questions above. You may also include additional comments for the author, including concerns about dual publication, research ethics, or publication ethics. (Please upload your review as an attachment if it exceeds 20,000 characters)  Reviewer #2: The authors have successfully incorporated the recommendations suggested by the reviewers. The work is now clearer, more open, more self-critical and more transparent. The decision to make the supporting information of the study available to readers is appreciated. In sum, it is a rigorous work that makes a necessary and valuable methodological contribution to the development and scientific consolidation of the Delphi method. A minor comment: In the revised text the first necessary feature of a Delphi study "Experts are surveyed while typically preserving their anonymity" (line 24 of the first draft) has been deleted. I assume this is a mistake. In addition, both line 24 and line 135 of the corrected manuscript still refer to the five typical characteristics of Delphi studies, despite the fact that only four are included in the new version. As far as I am concerned, once this error has been corrected, the article is perfectly publishable. Thank you very much. | Thank you for the positive feedback and for pointing out the error which we missed. It has been corrected and we have reinserted the missing characteristic. |
